# Scion varieties and nitrogen levels affect carbon and nitrogen assimilation in apple via modulating rhizosphere microbial structure and function

**DOI:** 10.1093/hr/uhaf334

**Published:** 2025-12-03

**Authors:** Huanhuan Zhang, Wen Zhang, Dongdong Yao, Xujiao Li, Hossam Salah Mahmoud Ali, Jingshan Xi, Yingchi Liang, Fengyun Zhao, Songlin Yu, Kun Yu

**Affiliations:** Department of Horticulture, College of Agriculture, Shihezi University, Shihezi 832003, China; Key Laboratory of Special Fruits and Vegetables Cultivation Physiology and Germplasm Resources Utilization of Xinjiang Production and Construction Corps, Department of Horticulture, College of Agriculture, Shihezi University, Shihezi 832003, China; Institute of Fruits and Vegetables, Xinjiang Academy of Agricultural Sciences, Urumqi 830091, China; Institute of Fruits and Vegetables, Xinjiang Academy of Agricultural Sciences, Urumqi 830091, China; Department of Horticulture, College of Agriculture, Shihezi University, Shihezi 832003, China; Key Laboratory of Special Fruits and Vegetables Cultivation Physiology and Germplasm Resources Utilization of Xinjiang Production and Construction Corps, Department of Horticulture, College of Agriculture, Shihezi University, Shihezi 832003, China; Institute of Fruits and Vegetables, Xinjiang Academy of Agricultural Sciences, Urumqi 830091, China; Department of Horticulture, College of Agriculture, Shihezi University, Shihezi 832003, China; Key Laboratory of Special Fruits and Vegetables Cultivation Physiology and Germplasm Resources Utilization of Xinjiang Production and Construction Corps, Department of Horticulture, College of Agriculture, Shihezi University, Shihezi 832003, China; Department of Horticulture, College of Agriculture, Shihezi University, Shihezi 832003, China; Key Laboratory of Special Fruits and Vegetables Cultivation Physiology and Germplasm Resources Utilization of Xinjiang Production and Construction Corps, Department of Horticulture, College of Agriculture, Shihezi University, Shihezi 832003, China; Department of Horticulture, College of Agriculture, Shihezi University, Shihezi 832003, China; Key Laboratory of Special Fruits and Vegetables Cultivation Physiology and Germplasm Resources Utilization of Xinjiang Production and Construction Corps, Department of Horticulture, College of Agriculture, Shihezi University, Shihezi 832003, China; Department of Horticulture, College of Agriculture, Shihezi University, Shihezi 832003, China; Key Laboratory of Special Fruits and Vegetables Cultivation Physiology and Germplasm Resources Utilization of Xinjiang Production and Construction Corps, Department of Horticulture, College of Agriculture, Shihezi University, Shihezi 832003, China; Department of Horticulture, College of Agriculture, Shihezi University, Shihezi 832003, China; Key Laboratory of Special Fruits and Vegetables Cultivation Physiology and Germplasm Resources Utilization of Xinjiang Production and Construction Corps, Department of Horticulture, College of Agriculture, Shihezi University, Shihezi 832003, China; Department of Horticulture, College of Agriculture, Shihezi University, Shihezi 832003, China; Key Laboratory of Special Fruits and Vegetables Cultivation Physiology and Germplasm Resources Utilization of Xinjiang Production and Construction Corps, Department of Horticulture, College of Agriculture, Shihezi University, Shihezi 832003, China; Department of Horticulture, College of Agriculture, Shihezi University, Shihezi 832003, China; Key Laboratory of Special Fruits and Vegetables Cultivation Physiology and Germplasm Resources Utilization of Xinjiang Production and Construction Corps, Department of Horticulture, College of Agriculture, Shihezi University, Shihezi 832003, China

## Abstract

The efficiency of carbon and nitrogen uptake in apple trees is co-regulated by plant genotype and rhizosphere microbial communities. However, the mechanisms by which different scion varieties modulate microbial structure and function under varying nitrogen levels remain poorly understood. In this study, *Malus sieversii* was used as the rootstock, onto which three scion cultivars (*M. sieversii*, *Malus domestica* cv. Hanfu, and *Malus domestica* cv. Red Fuji) were grafted under two nitrogen regimes. A combination of ^13^C/^15^N isotope labeling, Illumina MiSeq amplicon sequencing, and metagenomic analysis was employed to elucidate how scion–rootstock interactions and nitrogen availability affect carbon and nitrogen acquisition. Under nitrogen-deficient conditions, Red Fuji exhibited stronger root activity and larger root surface area, indicating enhanced nutrient foraging capacity. Conversely, under nitrogen application, Hanfu showed significantly greater ^13^C and ^15^N uptake, with 5.7-fold and 1.6-fold higher ^13^C accumulation in roots and stems, respectively, and markedly higher ^15^N utilization efficiency in roots and leaves compared with *M*. *sieversii*. In parallel, Hanfu under nitrogen input showed enrichment of beneficial microbial taxa and more complex microbial co-occurrence networks. Metagenomic analysis and random forest analyses revealed that the relative abundance of specific functional genes related to carbon and nitrogen transformation (*rbcL*, *abfA*, *napB*/*C*, *nasA*) was significantly higher under specific scion–nitrogen combinations, contributing to enhanced microbial carbon fixation and nitrogen reduction. Collectively, these results demonstrate that scion genotype modulates rhizosphere microbial structure, physiological root traits, and carbon–nitrogen distribution patterns, thereby improving nutrient uptake efficiency under different nitrogen inputs.

## Introduction

Apple (*Malus* × *domestica* Borkh.), a major perennial fruit crop cultivated worldwide, has demonstrated stable economic returns and industrial resilience over the past two decades [1]. During the growth and development of apple trees, the transport and allocation of photosynthates follow a typical ‘source–sink’ dynamic, whereby carbohydrates produced via photosynthesis are preferentially distributed to active growth centers and proximal tissues [2]. Nitrogen (N), an essential constituent of proteins, nucleic acids, and chlorophyll, plays a crucial role in regulating source–sink relationships, promoting dry matter accumulation, and improving fruit quality [3]. Different scion cultivars exhibit diverse physiological responses to N availability, reflecting their genetic variation in nutrient use efficiency. Identifying high-efficiency cultivars and understanding their nutrient regulation mechanisms is vital for optimizing fertilizer inputs and promoting sustainable apple production.

Grafting is a traditional yet widely adopted horticultural technique that holds immense importance in the cultivation of perennial woody fruit crops, such as apple, peach (*Prunus persica* (L.) Batsch), and grapevine (*Vitis vinifera* L.) [4]. By combining superior rootstocks with scions exhibiting specific advantages, grafting produces composite plants with strengthened morphological and physiological complementarity. This integration facilitates the efficient transport and redistribution of photosynthates, nutrients, and hormones within the plant [5]. Scions' physiological properties can exert considerable influence on root system architecture, functionality, and nutrient uptake capacity by modulating source–sink dynamics, altering sugar metabolite fluxes, and regulating hormone signaling pathways [6]. Recent studies have shown that different scion varieties can alter the N uptake kinetics of rootstocks, subsequently affecting carbohydrate accumulation and sugar metabolism characteristics in fruits [7]. However, there is a dearth of research investigating how scions modulate rootstock carbon (C) and N metabolic processes through their effects on rhizosphere microbial communities. Stable isotope tracing using ^13^C and ^15^N offers a robust approach for dissecting the transport and allocation of C and N within plant systems, soil nutrient transformations, and plant–microbe interactions [8, 9]. By accurately tracking isotope flows, it is possible to quantify the distribution patterns of C and N among different organs and reveal the regulatory mechanisms through which scion–rootstock interactions influence plant metabolism.

N fertilization exerts substantial positive effects on plant growth and nutrient accumulation, primarily through its capacity to improve soil physicochemical properties and reshape the soil biological environment [10]. Beyond increasing the availability of essential nutrients, N inputs indirectly modulate root nutrient uptake and metabolic processes by altering soil pH, organic matter content, and microbial activity [11]. N availability also regulates rhizosphere microbial communities, which in turn drive key biogeochemical processes, such as N fixation, nitrification, and denitrification [12]. By modifying microbial activity and composition, N influences C and N cycling and thus affects nutrient mineralization and uptake efficiency [13]. The rhizosphere, a specific microecological interface between plant roots and soil, harbors abundant active microbial communities that form microbial hotspots centered around root activity [14]. These rhizosphere microbes are central to driving the biogeochemical cycles of C, N, phosphorus, and sulfur, and they directly contribute to plant nutrient status and growth by promoting nutrient mineralization, facilitating transformation processes, and synthesizing plant growth-promoting substances [15]. Increasing evidence has also highlighted the significant role of rootstocks in influencing rhizosphere microbial community diversity, functional zonation, and niche partitioning by modulating root exudate composition, adjusting rhizosphere physicochemical conditions, and interacting with specific microbial taxa [16, 17]. Rootstock–scion combinations further affect microbial structure and activity, with scion genotypes shaping root exudation patterns and microbial interactions that impact C assimilation and N use efficiency [18]. While the effects of N fertilization on microbial communities are well documented, less is known about how different scion genotypes interact with N levels to shape rhizosphere microbiomes and affect C and N assimilation. Addressing this knowledge gap is essential for improving orchard management and sustainability, as understanding scion–N interactions can help optimize fertilization strategies and enhance nutrient use efficiency in apple production.

In this study, we hypothesized that scion varieties modulate rhizosphere microbial community structure and function, influencing C and N uptake in apple plants, and that these effects are regulated by N availability. To test this, *Malus sieversii* was used as the rootstock, grafted with three scion cultivars (*M*. *sieversii*, Hanfu, and Red Fuji) under two N regimes (no-N and N-fertilized). We combined ^13^CO_2_ pulse labeling, ^15^N tracing, amplicon sequencing, and metagenomics to address: (i) how scion genotype affects C and N assimilation under variable N conditions, and (ii) whether these effects are linked to changes in rhizosphere microbial composition and functional gene expression. Our findings aim to elucidate how scion–N interactions co-regulate rhizosphere microbiomes and contribute to nutrient use efficiency in apple, offering a foundation for microbiome-informed orchard management strategies.

## Results

### Response of apple growth and root morphology to different scion varieties and nitrogen levels

**Figure 1:**
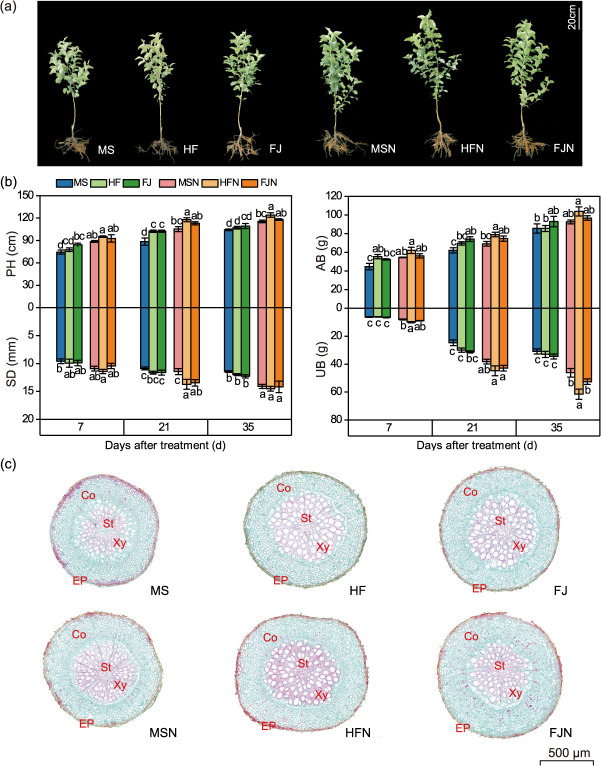
Nitrogen application promotes growth and root anatomical development in apple plants grafted with different scion varieties. (a) Phenotypic traits of apple seedlings; (b) PH, SD, AB, and UB; (c) root cross-sections imaged at 6 × magnification (scale bars = 500 μm). Anatomical labels: Co, cortex; St, stele; Xy, xylem; Ep, epidermis. Values represent means ± SD (*n* = 3). Different lowercase letters above bars indicate significant differences among treatments within the same period based on Tukey's honestly significant difference test (*P* < 0.05). MS grafted onto *M. sieversii*; HF grafted onto *M. sieversii*; FJ grafted onto *M. sieversii.* N represents nitrogen application treatment.

Both scion variety and N level significantly affected apple plant growth, root morphological traits, and root tip anatomical structure (Fig. 1; Figs S2 and S3; Tables S1–S3). On Day 35 following N treatment, under N-deficient (N0) treatment, Red Fuji (FJ) exhibited markedly greater root surface area (RS), root average diameter (RAD), root tip number (RT), and root activity (RA) than *M. sieversii* (MS), with respective increase of 20.18%, 23.62%, 33.06%, and 23.15%. Under N addition (N1) treatment, Hanfu (HF) showed significantly higher belowground biomass (UB), total biomass (TB), RS, RAD, RT, and RA than both MS and FJ. In particular, compared with MS, HF under N1 treatment displayed 7.47% and 29.96% increase in plant height (PH) and root length (RL), respectively. Root anatomical observations further revealed that N application significantly promoted structural development in HF roots. Specifically, HF exhibited larger cross-sectional area (CSA), stele diameter (STD), stele area (STA), and xylem area (XYA) than both MS and FJ. Moreover, root tip diameter (DI, anatomy), epidermis thickness (EPT), and vessel area (VEA) in HF were 10.66%, 10.46%, and 20.09% greater than those in MS, respectively. Correlation analysis indicated that CSA, VEA, RL, RS, and RA were all significantly positively correlated with aboveground biomass (AB), UB, and TB (Fig. S6).

### Effects of scion varieties and nitrogen levels on ^13^C and ^15^N uptake and distribution in apple

**Figure 2:**
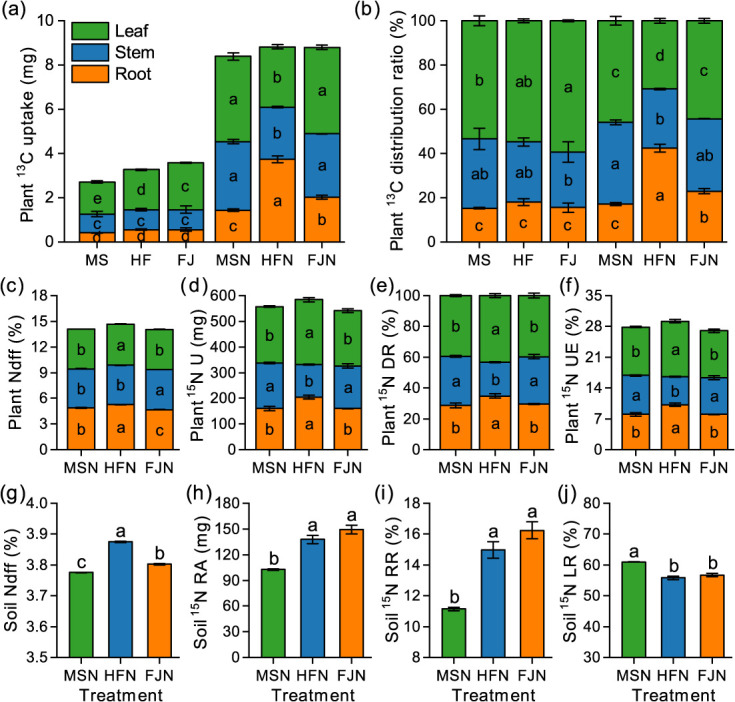
Uptake and distribution of ^13^C and ^15^N in different apple plant organs under different scion varieties and nitrogen levels. (a) Plant ^13^C uptake; (b) plant ^13^C distribution ratio; (c) plant Ndff; (d) plant ^15^N uptake (^15^N U); (e) plant ^15^N distribution ratio (^15^N DR); (f) plant ^15^N utilization efficiency (^15^N UE); (g) soil Ndff; (h) soil ^15^N residual amount (^15^N RA); (i) soil ^15^N residual rate (^15^N RR); (j) soil ^15^N loss ratio (^15^N LR). Values represent means ± SD (*n* = 3). Different lowercase letters on bars indicate significant differences among treatments within the same organ (leaf, stem, or root) based on Tukey’s honestly significant difference test (*P* < 0.05). MS grafted onto *M. sieversii*; HF grafted onto *M. sieversii*; FJ grafted onto *M. sieversii.* N represents nitrogen application treatment.

N application significantly promoted ^13^C uptake and its distribution across all plant organs (Fig. 2a; Fig. S4; Table S3). Compared with the N0 treatment, ^13^C uptake by roots, stems, and leaves in HF under N1 treatment increased by 5.72-fold, 1.61-fold, and 0.50-fold, respectively. Under N0 treatment, both HF and FJ showed higher ^13^C uptake and relative distribution in leaves compared to MS. Under N1 treatment HF demonstrated a 161.03% increase in root ^13^C uptake and a 34.21% increase in its distribution compared to MS. Correlation analysis revealed that ^13^C uptake in leaves, stems, and roots was significantly positively correlated with AB, UB, and TB (Fig. S6). For ^15^N dynamics, HF displayed significantly higher values of Ndff, ^15^N uptake, ^15^N distribution, and ^15^N utilization rates in both leaves and roots compared to MS and FJ under N1 treatment (Fig. 2c–j; Fig. S5). In particular, the ^15^N utilization rate in HF roots was 27.13% and 26.99% higher than that in MS and FJ, respectively. Furthermore, under N1 treatment, HF and FJ had markedly higher soil ^15^N retention rates than MS by 34.45% and 45.79%, while their soil ^15^N loss rates were significantly lower than MS by 8.48% and 7.00%, respectively.

### Changes in rhizosphere microbial community diversity and structure under different scion varieties and nitrogen levels

**Figure 3:**
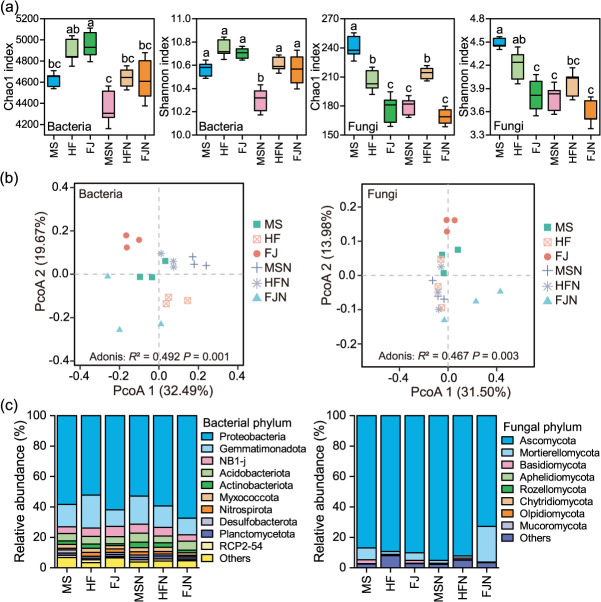
Diversity and structure of rhizosphere bacterial and fungal communities under different scion varieties and nitrogen levels. (a) Alpha diversity indices (Chao1 and Shannon); (b) principal coordinates analysis (PCoA) based on Bray-Curtis distance; (c) relative abundances of dominant bacterial and fungal phyla. Values represent means ± SD (*n* = 3). Different lowercase letters above bars indicate significant differences among treatments within the same period based on Tukey’s honestly significant difference test (*P* < 0.05). MS grafted onto *M. sieversii*; HF grafted onto *M. sieversii*; FJ grafted onto *M. sieversii.* N represents nitrogen application treatment.

High-throughput sequencing of the 16S rRNA V3–V4 and ITS regions yielded a total of 2 502 526 and 2 471 864 raw reads, respectively (Tables S4 and S5). Following quality filtering, 770 132 high-quality bacterial sequences and 2 119 621 high-quality fungal sequences were retained, from which 1091 bacterial and 86 fungal amplicon sequence variants (ASVs) were identified (Fig. S7). Alpha diversity analysis showed that N addition led to a reduction in both Chao1 and Shannon indices for bacterial and fungal communities across all three scion combinations (Fig. 3a). Regardless of N level, HF and FJ consistently exhibited higher bacterial community diversity than MS. In contrast, fungal community diversity patterns varied with N availability: under N0 treatment, diversity followed the order MS > HF > FJ, whereas under N1 treatment, the trend shifted to HF > MS > FJ. Principal coordinates analysis (PCoA) further demonstrated that both scion genotype and N level exerted significant effects on the rhizosphere bacterial (Adonis *R*^2^ = 0.492, *P* = 0.001) and fungal (Adonis *R*^2^ = 0.467, *P* = 0.003) community structures (Fig. 3b).

At the phylum level, Proteobacteria (52.14%–67.38%) and Gemmatimonadota (10.90%–21.82%) were the dominant bacterial taxa across all treatments (Fig. 3c). Under N0 treatment, the relative abundance of Proteobacteria in HF was significantly lower, while that of Gemmatimonadota was significantly higher, compared to MS. The opposite trend was observed under N1 treatment (Table S6). For fungi, Ascomycota (72.87%–95.18%) and Mortierellomycota (1.56%–23.31%) were the predominant phyla. Under N0 treatment, MS harbored a significantly higher relative abundance of Mortierellomycota than HF; under N1 treatment, FJ had significantly higher Mortierellomycota levels than both MS and HF. At the genus level, under N0 treatment, FJ was enriched in *Ellin6067*, *Sphingomonas*, *Dongia*, and *Nitrospira* compared to MS and HF. In contrast, under N1 treatment, HF had significantly higher relative abundances of *SWB02*, RCP2–54, and *Haliangium*, but a markedly reduced abundance of *Sphingomonas* (with a 75.65% decrease relative to FJ) (Fig. S8; Table S7). For fungal genera, HF under N0 treatment showed higher relative abundances of *Doratomyces* and *Aspergillus* than MS and FJ. Following N application, HF exhibited 50.98% and 77.90% higher relative abundances of *Doratomyces* and *Schizothecium*, respectively, compared to FJ; conversely, the abundances of *Botryotrichum*, *Mortierella*, and *Fusarium* were significantly lower in HF (Fig. S8; Table S8).

Co-occurrence network analysis showed that microbial interaction patterns were jointly modulated by N availability and scion genotype. Under N1 treatment, the rhizosphere bacterial and fungal networks associated with HF exhibited the highest numbers of nodes, edges, average degree, and network density (Fig. S9; Table S9). In contrast, under N0 treatment, MS presented larger and denser networks, whereas HF showed a higher proportion of positive correlations among microbial taxa.

### Importance of rhizosphere microorganisms in ^13^C and ^15^N uptake based on random forest modeling

Random forest modeling revealed that the diversity, network properties, and dominant genera of rhizosphere bacterial and fungal communities were key determinants of ^13^C and ^15^N uptake in apple plants (Fig. S10). At the genus level, nine bacterial and nine fungal genera were identified as the key predictors of total ^13^C uptake and total ^15^N uptake across plant organs. Among the top predictors of total plant ^13^C uptake (T^13^CU) were *Schizothecium* (8.61%), *Gemmatimonas* (5.13%), *Doratomyces* (5.08%), and *Sphingomonas* (3.78%); in contrast, total plant ^15^N uptake (T^15^NU) was most strongly predicted by *RCP2–54* (6.41%), *Mortierella* (4.20%), *Doratomyces* (4.06%), and *Haliangium* (3.99%) (Fig. S11). Further correlation analysis showed that T^13^CU was positively associated with the relative abundances of *mle1*–*7* and *Schizothecium*, but negatively correlated with *Gemmatimonas* and *Cephalotrichum* (Fig. S12a). Likewise, T^15^NU showed significant positive correlations with *SWB02*, *Vicinamibacteraceae*, *OM190*, *RCP2*–*54*, and *Doratomyces*, while being negatively correlated with *Ellin6067*, *Sphingomonas*, *Haliangium*, *Mortierellomycota*, *Botryotrichum*, and *Mortierella* (Fig. S12b).

### Correlations between plant/environmental variables and potentially beneficial microorganisms

**Figure 4:**
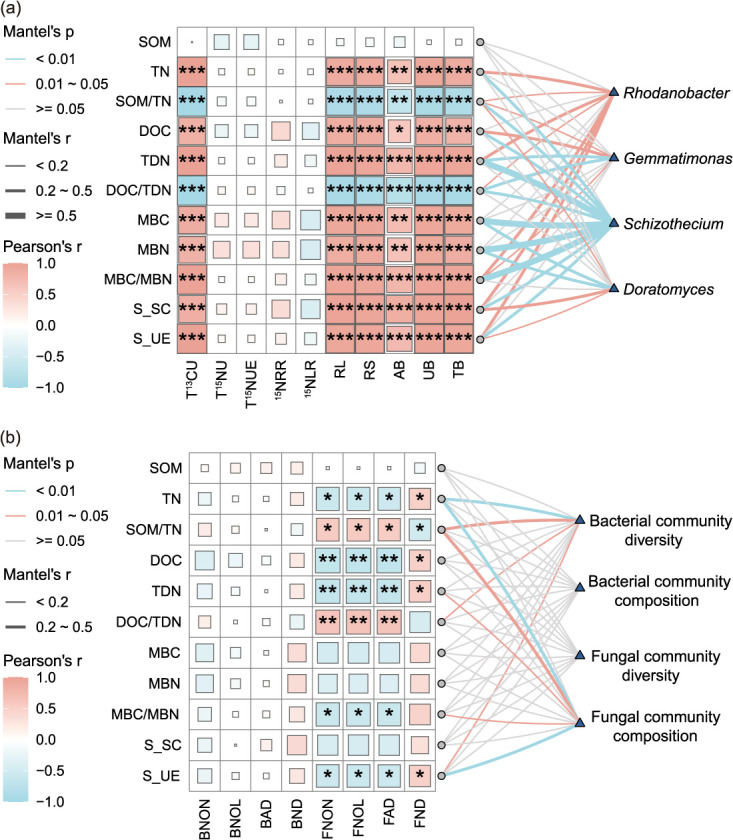
Correlation networks among environmental, plant, and microbial factors. Correlation networks between environmental/plant variables and (a) beneficial microbes, and (b) microbial community diversity and composition and network topological features. Colors indicate the direction of correlation (positive or negative). Edge width corresponds to the Mantel's *r* value determined by linear mixed-effects models. Solid and dashed lines denote significant and nonsignificant correlations, respectively. Pairwise comparisons of factors are displayed in the rectangle, with a color gradient denoting Pearson's correlation coefficients. ^*^P < 0.05, ^**^P < 0.01, ^***^P < 0.001. S_SC, soil SC; S_UE, soil UE. T^13^CU, total plant ^13^C uptake; T^15^NU, total plant ^15^N uptake; T^15^NUE, total plant ^15^N utilization efficiency; ^15^NRR, ^15^N residual rate; ^15^NLR, ^15^N loss ratio; RL, root length; RS, root surface area; BNON, number of nodes in the bacterial community network; BNOL, number of links in the bacterial community network; BAD, average degree in the bacterial community network; BND, density in the bacterial community network; FND, density in the fungal community network.

Scion variety and N level significantly affected the physicochemical and enzymatic properties of rhizosphere soil (Table S10). Compared with the N0 treatment, the N1 treatment significantly increased total N (TN), total dissolved N (TDN), microbial biomass C (MBC), microbial biomass N (MBN), and the activities of soil sucrase (SC) and soil urease (UE) while decreasing the soil organic matter-to-N ratio (SOM/TN) and dissolved organic C-to-N ratio (DOC/TDN). Under N0 treatment, no significant differences were observed among the scion varieties in terms of TN and DOC contents, or SC and UE activities. However, under N1 treatment, HF exhibited significantly higher TDN, MBC, MBN, MBC/MBN, and UE activity than MS. Correlation analysis disclosed that TN, DOC, TDN, MBC, MBN, MBC/MBN, SC, and UE were positively correlated with T^13^CU, RL, RS, AB, UB, and TB, but negatively associated with SOM/TN and DOC/TDN (Fig. 4a). Beneficial microbial genera such as *Rhodanobacter* and *Schizothecium* were significantly associated with SOM/TN, TDN, DOC/TDN, MBC/MBN, SC, and UE. Fungal network properties, including the number of nodes (FNON), number of links (FNOL), and average degree (FAD), were positively associated with SOM/TN and DOC/TDN, but negatively associated with TN, DOC, and TDN (Fig. 4b). Moreover, the diversity of bacterial communities and the composition of fungal communities were significantly related to TN, SOM/TN, and UE.

### Functional gene responses in rhizosphere carbon and nitrogen cycling to scion varieties and nitrogen levels

**Figure 5:**
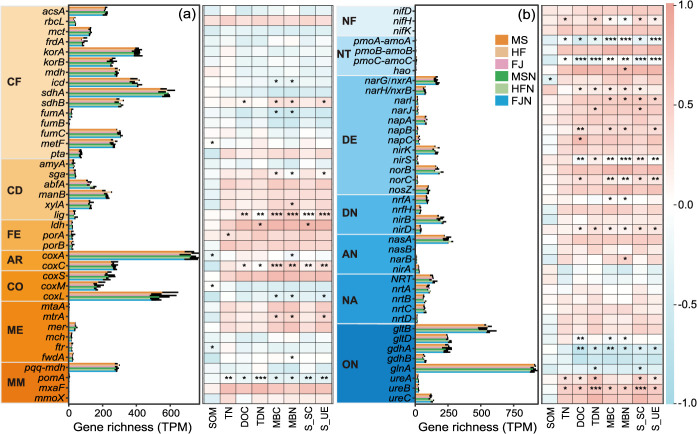
Functional gene abundances related to rhizosphere C and N cycling. Relative abundances of key functional genes involved in microbial (a) C and (b) N cycling in the rhizosphere of apple plants under different scion varieties and nitrogen levels, along with their correlations with rhizosphere soil properties. Pairwise comparisons of factors are displayed in the rectangle, with a color gradient denoting Spearman’s correlation coefficients. ^*^P < 0.05, ^**^P < 0.01, ^***^P < 0.001. CF, carbon fixation; CD, carbon degradation; FE, fermentation; AR, aerobic respiration; CO, CO oxidation; ME, methanogenesis; MM, methane metabolism; NF, N_2_ fixation; NT, nitrification; DE, denitrification; DN, dissimilatory nitrate reduction to ammonium; AN, assimilatory nitrate reduction to ammonium; NA, nitrate assimilation; ON, organic nitrogen metabolism; S_SC, soil SC; S_UE, soil UE.

Microbial functional gene analysis revealed clear differences in C and N cycling among scion varieties under different N treatments (Fig. 5). In the C cycling, the main metabolic pathways included C fixation (CF), C degradation (CD), and aerobic respiration (AR) (Fig. 5a). Under N1 treatment, HF exhibited higher relative abundances of genes related to CF (e.g. *rbcL*, *icd*, *sdhB*, *fumC*), CD (e.g. *amyA*, *sga*, *abfA*, *lig*), and AR (e.g. coxA) compared to MS and FJ. Under N0 treatment, however, HF showed reduced abundances of CF-related genes (*rbcL*, *mct*, *frdA*, *korB*, *pta*) compared to MS and FJ, but elevated levels of *korA*, *mdh*, *icd*, *sdhA*/*B*/*C*, and *metF* (Table S11). Most CF-related genes were negatively correlated with SOM content. MBC and MBN levels were negatively correlated with CF genes but positively associated with CD genes such as *aga* and *lig*, and the AR gene *coxC*.

In the N cycling, genes related to organic N metabolism (ON) were the most abundant, while those associated with N fixation (NF) and nitrification (NT) appeared at lower abundances (Fig. 5b). Under N1 treatment, HF exhibited higher relative abundances of NF (*nifH*, *nifK*) and denitrification (DE) genes (*narG*/*nxrA*, *narH*/*nxrB*, *narJ*, *napA*, *nirK*, *norB*/*C*) compared to MS and FJ, but lower abundances of NT genes (*pmoA*-*amoA*, *pmoB*-*amoB*, *pmoC*-*amoC*) (Fig. S13; Table S12). Soil TN and TDN were positively correlated with NF genes (*nifH* and *ureA*/*B*), while DOC, TDN, MBC, MBN, SC, and UE were also positively correlated with *ureB* but negatively correlated with *pmoA*-*amoA* and *pmoC*-*amoC*. Random forest modeling identified key predictive genes for T^13^CU, including *rbcL*, *abfA*, *manB*, and ldh, and for T^15^NU, including *napB*/*C* and *nasA* (Fig. S14). Redundancy analysis (RDA) indicated that TDN and MBC were the main environmental factors influencing dominant microbial genera, whereas MBN and SC primarily affected functional gene variation related to C and N cycling (Fig. S15; Table S13).

## Discussion

### Response of apple growth to different scion varieties and nitrogen levels

As widely reported, N fertilization significantly enhances leaf photosynthetic capacity and assimilate accumulation, ultimately contributing to increased biomass and crop yield [3, 19]. In this study, N addition notably improved PH, stem diameter (SD), and biomass accumulation across all scion cultivars, demonstrating enhanced N uptake and utilization efficiency. However, the magnitude of these growth responses varied substantially among scion types, highlighting genotype-dependent differences in growth strategies and nutrient allocation. Specifically, under N fertilization, Hanfu exhibited the greatest increases in PH, UB, and root development compared to *M*. *sieversii* and Red Fuji, suggesting superior N use efficiency in Hanfu. This finding is consistent with previous studies that show N-efficient cultivars tend to exhibit elevated nitrate reductase and glutamine synthetase activities, enhancing N assimilation [20]. In contrast, Red Fuji demonstrated greater RS and root activity under N-deficient conditions, suggesting a compensatory strategy aimed at enhancing nutrient foraging under low N availability. These observations indicate that Red Fuji may rely on adaptive root traits such as increased root tip number and enhanced root hair density to acquire N when nutrient levels are limited [21]. However, when N supplementation was provided, Hanfu outperformed Red Fuji, illustrating that the two cultivars adopt divergent strategies for N utilization. Moreover, strong positive correlations between root anatomical traits (CSA and VEA) and shoot/root/TB emphasize the importance of root anatomical optimization in supporting aboveground growth. This root anatomical development likely plays a role in enhancing nutrient and water uptake, which, in turn, creates a favorable environment for microbial communities involved in nutrient cycling [18].

### Effects of scion varieties and nitrogen levels on carbon and nitrogen uptake

N fertilization boosts N uptake and facilitates the photosynthates synthesis and translocation, thereby improving overall N use efficiency [22, 23]. Previous studies have shown that appropriate N supplementation not only accelerates plant growth but also improves photosynthetic capacity and the uptake of other nutrients by optimizing N assimilation pathways [23, 24]. In our study, N application significantly increased both ^13^C and ^15^N uptake in Hanfu, which exhibited the highest levels of ^15^N utilization efficiency in roots and leaves. Under N fertilization, the ^13^C uptake by roots in Hanfu increased by 5.72-fold compared to the no-N control, reflecting enhanced root C metabolism and a more favorable rhizosphere environment for N assimilation. These findings highlight the critical role of the rhizosphere environment in facilitating plant nutrient acquisition, with microbial activity playing a key role in N cycling [25]. Prior research has demonstrated that rhizosphere microbial composition can profoundly affect soil N transformation and availability [22, 26]. In our study, the increased ^13^C uptake observed in Hanfu roots suggests a role in enhanced C metabolic activity, likely contributing to a rhizosphere priming effect. This priming effect is supported by increased enzyme activities, such as SC and UE, which promote the breakdown of organic matter and microbial necromass, accelerating N mineralization and increasing plant-available N [27]. The higher ^15^N retention and lower ^15^N loss in the rhizosphere soil of Hanfu further support its superior N assimilation capacity under N fertilization. In contrast, Red Fuji adopted a different C allocation strategy by preferentially distributing ^13^C to stems under N fertilization. This may reflect a cultivar-specific ‘source–sink’ regulation mechanism typical of high-yielding, late-maturing apple varieties, in which the stems function as temporary C sinks to support later-stage fruit development [28]. Nevertheless, this allocation pattern may reduce C inputs to roots, potentially limiting N uptake efficiency [29]. Therefore, Red Fuji may prioritize aboveground C sinks over root C supply under sufficient N conditions, which could impact its N use dynamics.

### Microbiome-mediated enhancement of carbon and nitrogen uptake under different scion and nitrogen treatments

Rhizosphere microbial community diversity is generally associated with greater ecosystem stability, enabling plants to better cope with environmental fluctuations [30]. This resilience is often attributed to functional redundancy and complementarity among microbial taxa, which together ensure the continuity of key ecological processes such as nutrient cycling, organic matter decomposition, and plant–microbe interactions [31]. In this study, however, N fertilization led to a reduction in both bacterial and fungal community diversity in the rhizosphere, suggesting that N inputs may suppress sensitive microbial groups. This suppression is likely driven by changes in rhizosphere C:N ratios, shifts in pH, and the dominance of copiotrophic microbes [32, 33]. These findings echo growing concerns that while N inputs can enhance short-term nutrient acquisition, they may also induce long-term ecological trade-offs by reducing microbial community diversity and potentially compromising soil functionality. Interestingly, despite the overall decline in diversity with N addition, both Hanfu and Red Fuji maintained significantly higher rhizosphere microbial community diversity than *M*. *sieversii*, likely due to genetic differences in scion-induced modulation of root exudates and C allocation [34, 35]. At the phylum level, Proteobacteria and Gemmatimonadota dominated the bacterial communities, while Ascomycota and Mortierellomycota were the main fungal taxa. Under N-deficient conditions, Hanfu showed a lower relative abundance of Proteobacteria but higher abundance of Gemmatimonadota compared to *M*. *sieversii*, suggesting a shift towards taxa better adapted to C:N fluctuations [36, 37]. Conversely, N addition reversed this pattern by promoting the proliferation of fast-growing, C-demanding Proteobacteria, likely in response to increased availability of root-derived C. In the meanwhile, Gemmatimonadota abundance declined possibly because of intensified resource competition [38]. Under N supplementation, however, Red Fuji retained a high abundance of Mortierellomycota, indicating a relatively weak response to N input. In contrast, Hanfu enriched *Doratomyces* and *Schizothecium* and suppressed potential pathogens like *Fusarium* and *Botryotrichum*. These taxa likely enhance soil N availability via organic matter degradation and simultaneously reduce N losses linked to pathogen-driven volatilization or leaching [39, 40]. At the genus level, *Schizothecium*, *Gemmatimonas*, and *Doratomyces* were identified as key predictors of total ^13^C uptake. Among them, *Schizothecium* and *Doratomyces* were positively associated with ^13^C accumulation, suggesting their roles in enhancing C assimilation, whereas *Gemmatimonas* showed a negative correlation, potentially reflecting its competition for C or its C-consuming role. Similarly, *RCP2–54*, *Haliangium*, and *Mortierella* were major contributors to total ^15^N uptake, with *Mortierella* exhibiting a negative association possibly due to its competition for N resources [41, 42]. Moreover, co-occurrence network analysis revealed that Hanfu constructed a more complex and interconnected rhizosphere microbial community under N fertilization, as evidenced by greater node number, edge density, and interaction strength. This structural complexity indicates robust microbial ecological resilience and stronger functional coordination [43]. Notably, even under N-deficient conditions, Hanfu maintained a high proportion of positive microbial interactions, suggesting sustained metabolic synergy that supports nutrient acquisition under stress. These findings suggest a role of scion genotype in modulating rhizosphere microbiomes, which may contribute to improved nutrient assimilation.

### Scion and nitrogen-level driven modulation of rhizosphere carbon and nitrogen cycling

**Figure 6:**
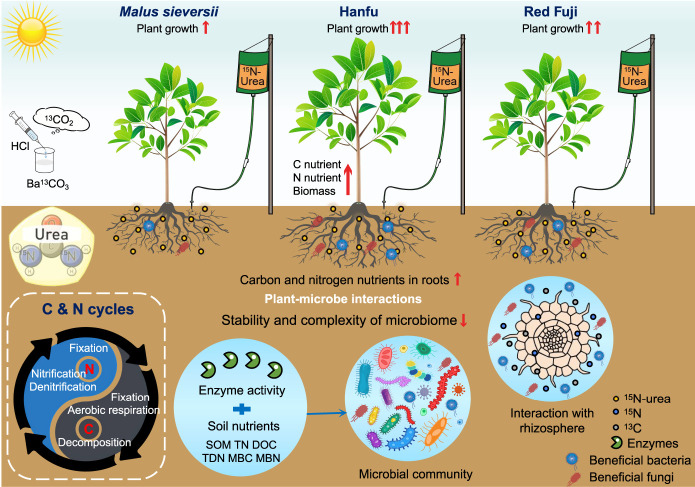
Conceptual model of scion and nitrogen effects on rhizosphere processes. Schematic diagram illustrating the processes and potential mechanisms by which scion genotype and nitrogen input regulate rhizosphere C and N cycling and thus plant growth in apple. C, carbon; N, nitrogen; SOM, soil organic matter; TN, total nitrogen; DOC, dissolved organic carbon; TDN, total dissolved nitrogen; MBC, microbial biomass carbon; MBN, microbial biomass nitrogen.

C and N cycling are fundamental governing soil nutrient balance and plant nutrient acquisition, largely mediated by plant–microbe interactions [44]. Our metagenomic analyses revealed Hanfu under N fertilization activated microbial pathways involved in both C fixation and N reduction. This was evidenced by an increase in the relative abundances of genes associated with C fixation (e.g. *rbcL*, *icd*, *sdhB*, *fumC*) and aerobic respiration (*coxA*), indicating stimulated microbial activity fueled by enhanced plant photosynthesis [45]. *RbcL* is pivotal for microbial-mediated C fixation, directly influencing the microbial capacity to sequester atmospheric CO_2_ into organic forms, which enhances soil fertility and plant growth in orchard ecosystems [46]. Interestingly, under N-deficient conditions, the expression of *rbcL* declined, while TCA-cycle genes (e.g. *sdhA*/*B*, *icd*) were upregulated, reflecting microbial adaptation to C limitation [47]. Regarding N cycling, Hanfu exhibited upregulation of N-fixing genes (*nifH*, *nifK*) and denitrification genes (*narG*, *napA*, *nirK*), alongside a suppression of nitrification genes (*pmoA-amoA*), highlighting a a preference for reductive N pathways to minimize nitrate leaching [48]. Similarly, genes involved in nitrate reduction pathways, such as *napB*/*C* and *nasA*, regulate nitrate transformation processes in the rhizosphere [48]. These pathways influence soil N dynamics by promoting denitrification, which reduces N loss and enhances N retention in the soil. Moreover, key N-related genes (e.g. *ureA*/*B*) were positively correlated with soil UE activity, TDN, and MBN, indicating microbially driven N transformation. Importantly, random forest modeling identified *rbcL*, *abfA*, *manB*, and *ldh* as strong predictors of ^13^C uptake, emphasizing the vital value of C fixation and carbohydrate metabolism. In the meanwhile, *napB*/*C* and *nasA* were found to be top predictors of ^15^N uptake, underscoring the significance of nitrate reduction and assimilation [49, 50]. RDA results further confirmed that rhizosphere TDN, MBC, and MBN were major environmental drivers shaping microbial community composition and functional gene expression. In summary, the Hanfu scion promoted C and N bioavailability under N fertilization by activating microbial pathways related to C fixation and N reduction, while simultaneously coordinating the expression of key microbial genes (Fig. 6). This process is contingent not only upon plant-driven root exudation but also on the rapid feedback of rhizosphere microbes to environmental cues [51]. Collectively, our findings advance the understanding of microecological mechanisms underlying nutrient regulation in perennial fruit tree systems and provide new insights into microbial-based strategies for enhancing nutrient use efficiency in orchard management.

## Materials and methods

### Plant materials and experimental design

The experiment was carried out in a full-steel arch plastic film greenhouse at the Experimental Station of the College of Agriculture, Shihezi University (45°19′N, 86°03′E). During the experimental period, the photoperiod for apple cultivation ranged from 12 h/12 h to 14 h/10 h (light/dark), with average day/night temperatures between 17°C and 35°C and relative humidity fluctuating between 35% and 60%. The test soil was sandy loam collected from the 0–20 cm surface layer of an apple orchard in Shihezi. Its physicochemical properties were as follows: organic matter, 18.44 g kg^−1^; total N, 1.21 g kg^−1^; available N, 32.38 mg kg^−1^; available phosphorus, 18.46 mg kg^−1^; available potassium, 125.75 mg kg^−1^; pH, 8.06; and total salt content, 3.75 g kg^−1^. Root-restricting polyvinyl chloride (PVC) pots (50 cm in diameter and 50 cm in height) were used, each filled with 100 kg of sieved, debris-free fresh soil. The experiment included three graft combinations: *M*. *sieversii* as the rootstock and scion (control treatment), *Malus domestica* cv. Hanfu, and *Malus domestica* cv. Red Fuji grafted onto *M*. *sieversii* rootstocks. Furthermore, *M*. *sieversii* is a species known for its resilience and stress tolerance, making it an ideal rootstock for studying the rootstock-scion interactions under varying environmental conditions [52]. The grafted saplings were transplanted centrally into each pot and uniformly pruned to a height of 60 cm.

N treatments commenced 63 days after transplantation. Urea (46% N) was applied as the N source at two levels: no N addition (0 g N kg^−1^ soil) and N addition (0.2 g N kg^−1^ soil), based on previous studies [53]. Each combination of N level and scion variety included six saplings, with three biological replicates per treatment, where each biological replicate corresponds to a single plant in a separate pot, resulting in a total of 108 grafted apple plants. For ^15^N labeling, a urea solution enriched with 10.16% ^15^N (prepared by dissolving 2 g of ^15^N-labelled urea and 41.48 g of regular urea in water) was slowly introduced into the root zone via infusion bags (Fig. S1). After 32 days of N treatment, ^13^C pulse labeling was performed by releasing ^13^CO_2_ from Ba^13^CO_3_ within a sealed transparent plastic chamber, maintaining labelling conditions for a duration of 4 h. Throughout the experimental period, all plants were irrigated using subsurface drip irrigation systems (5.0 L h^−1^ per emitter, applied once every 7 days). Growth conditions and cultivation management practices were kept consistent across all treatments.

### Plant height, stem diameter, and biomass

At 7, 21, and 35 days following N application, three uniformly growing saplings were randomly selected from each treatment group. PH was measured from the stem base to the shoot apex using a measuring tape, while SD was measured 5 cm above the graft union using a digital caliper. For biomass determination, an additional three saplings per treatment were harvested and separated into roots, stems, and leaves. Each organ was sequentially rinsed with tap water, detergent solution, 1% HCl, and finally deionized water. The samples were then blanched at 105°C for 30 min, followed by oven-drying at 80°C until a constant weight was achieved. Biomass of each organ was measured using an analytical balance (± 0.001 g), and the AB, UB, and TB were calculated accordingly.

### Root morphology and anatomical structure

On Day 35 after N treatment, fresh roots were rinsed with tap water followed by deionized water to remove adhering soil particles. For each treatment group, 3 trees were randomly selected, with each tree representing a biological replicate. Root morphological traits were assessed by scanning the cleaned roots using a root scanner (Expression 2400, EPSON, Japan), and the images were analyzed with WinRHIZO (Regent Instrument Inc., Canada). The following parameters were quantified: total root length (RL), RS, root volume (RV), RAD, and root tip number (RT). Root activity (RA) was determined by the 2,3,5-triphenyltetrazolium chloride reduction method.

To examine root tip anatomical structure, actively growing root segments were selected and dried with filter paper. Root tips (2–3 cm in length) were trimmed into 0.5 cm sections and fixed in FAA solution (formaldehyde-acetic acid-ethanol) at 4°C for 24 hours. The fixed samples were then dehydrated, embedded in paraffin, sectioned at a thickness of 8 to 10 μm, and stained sequentially with 1% safranin and 0.5% fast green. Sections were observed under a light microscope (BX51; Olympus, Japan), and a minimum of five visual fields per sample were analyzed using AxioVision 4.8 (Carl Zeiss, Beijing). Anatomical parameters included DI, CSA, EPT, cortex thickness (COT), vessel diameter (VED), VEA, STD, STA, and XYA.

### C and N analyses

On Day 35 after N treatment, three apple saplings were randomly selected from each treatment group. Rhizosphere soil adhering tightly to the RS was collected using sterile soft brushes and passed through a 100-mesh sieve. Roots, stems, and leaves from each plant were separately harvested, oven-dried, finely ground, and sieved through a 0.25-mm mesh. Total C (TC) and total N (TN) contents in plant organs and rhizosphere soil were determined using a C–N elemental analyzer (Elementar Vario EL, Elementar, Germany). The relative abundances of ^13^C and ^15^N (expressed as δ^13^C and δ^15^N) were measured with an isotope ratio mass spectrometer (DELTA V Advantage; Thermo Fisher Scientific Inc., USA).

Isotopic labeling parameters were calculated using the following formulas:


(1) \begin{equation*} \mathrm{\delta} (\%) =\left(\frac{\mathrm{R}\_\mathrm{sample}}{\mathrm{R}\_\mathrm{standard}}-1\right)\times1000 \end{equation*}

(2) \begin{equation*} \mathrm{Atom} (\%)=\left(\frac{\mathrm{R}\_\mathrm{sample}}{\mathrm{R}\_\mathrm{sample}+1}\right)\times100 \end{equation*}

(3) \begin{equation*}\text{C content in organ (g)} = \text{Biomass (g)}\times \mathrm{TC}\,(\%) \end{equation*}

(4) \begin{align*}\notag ^{13}\text{C absorbed (mg)}&= \text{C content} \times (\mathrm{Atom}\%^{13}\mathrm{C}_{sample}\\&\quad-\mathrm{Atom}\%^{13}\mathrm{C}_{natural})\times1000 \end{align*}

(5) \begin{equation*} \mathrm{Total}^{13}\text{C absorbed (mg)}=\, ^{13}\!\mathrm{C}_{leaf}+^{13}\!\mathrm{C}_{stem}+^{13}\!\mathrm{C}_{root} \end{equation*}

(6) \begin{equation*} ^{13}\!\text{C distribution ratio }(\%)=\left(\frac{{\mathrm{Organ}}^{13}\mathrm{C}}{{\mathrm{Total}}^{13}\mathrm{C}}\right)\times100 \end{equation*}

(7) \begin{equation*} \mathrm{Ndff}\,(\%)=\frac{\left(\mathrm{Atom}{\%}^{15}\mathrm{N}\_\mathrm{sample}\hbox{--} \mathrm{Atom}{\%}^{15}\mathrm{N}\_\mathrm{natural}\right)}{\left(\mathrm{Atom}{\%}^{15}\mathrm{N}\_\mathrm{fertilizer}\hbox{--} \mathrm{Atom}{\%}^{15}\mathrm{N}\_\mathrm{natural}\right)}\times100 \end{equation*}

(8) \begin{equation*} \text{N content in organ (g)}= \text{Biomass (g)}\times\mathrm{TN}\,(\%) \end{equation*}

(9) \begin{align*}^{15}\text{N absorbed (mg)}&=\text{N content}\,\times\, (\mathrm{Atom}\%^{15}\mathrm{N}_{sample}\notag\\&\quad-\mathrm{Atom}\%^{15}\mathrm{N}_{natural})\times1000 \end{align*}

(10) \begin{equation*} ^{15}\text{N distribution ratio}\,(\%)=\left(\frac{{\mathrm{Organ}}^{15}\mathrm{N}}{{\mathrm{Total}}^{15}\mathrm{N}}\right)\times100 \end{equation*}

(11) \begin{equation*} ^{15}\text{N utilization rate}\,(\%)=\left(\frac{{\mathrm{Plant}}^{15}\mathrm{N}\ \mathrm{absorbed}}{{\mathrm{Total}\ \mathrm{applied}}^{15}\mathrm{N}}\right)\times100 \end{equation*}

(12) \begin{equation*} \mathrm{Soil}\ ^{15}\text{N residual amount (g)} \!=\! \text{Soil dry weight (g)}\! \times \mathrm{TN}\ (\%)\times\mathrm{Ndff}(\%) \end{equation*}

(13) \begin{equation*} \mathrm{Soil}\ ^{15}\text{N retention rate} (\%) =\left(\frac{{\mathrm{Soil}}^{15}\mathrm{N}\ \mathrm{residual}\ \mathrm{amount}}{{\mathrm{Total}\ \mathrm{applied}}^{15}\mathrm{N}}\right)\times100 \end{equation*}

(14) \begin{equation*} {}^{15}\text{N loss rate} (\%)={100\%}-{}^{15}\!\text{N utilization rate}-{}^{15}\!\text{N retention rate} \end{equation*}

where δ^13^C and δ^15^N represent isotopic enrichment levels; R_sample is the isotope ratio (^13^C/^12^C or ^15^N/^14^N) in the sample; R_standard refers to standard isotope ratios (0.0112372 for C and 0.0036765 for N); Atom%^13^C_natural = 1.1078%, Atom%^15^N_natural = 0.3663%; Ndff represents the percentage of N derived from the applied fertilizer.

### Rhizosphere soil physicochemical properties and enzyme activities

Rhizosphere soil samples were passed through a 10-mesh sieve and stored at 4°C prior to the determination of physicochemical properties and enzyme activities. MBC and MBN were quantified using the chloroform fumigation–extraction method. Specifically, fumigated and non-fumigated soil samples were extracted with 0.5 mol∙L^−1^ K_2_SO_4_ solution. Organic C in the extracts was measured by the potassium dichromate oxidation method, while N content was determined via the Kjeldahl method. MBC and MBN were calculated using the following equations: MBC = Ec/0.38 and MBN = En/0.45, where Ec and En represent the differences in C and N contents between fumigated and control samples, respectively. TDN was determined via potassium persulfate oxidation followed by UV–Vis spectrophotometry (UV–Vis 2600, Shimadzu, Japan). DOC was defined as the organic C content in the non-fumigated soil extracts. SC activity (EC 3.2.1.26) was assessed using the 3,5-dinitrosalicylic acid colorimetric method and expressed as the amount of glucose released per gram of soil per day (mg∙g^−1^∙d^−1^). UE activity (EC 3.5.1.5) was determined using the phenol–sodium hypochlorite colorimetric method and reported as the amount of NH_4_^+^-N released per gram of soil per day (μg∙g^−1^∙d^−1^) [54].

### DNA extraction, amplicon sequencing, and bioinformatics

On Day 35 after N treatment, nine apple saplings were randomly selected from each treatment group. The rhizosphere soil from every three plants was mixed to form one composite sample, yielding three biological replicates per treatment. Approximately 10 g of each composite sample was used for DNA extraction with the E.Z.N.A.® Soil DNA Kit (Omega Bio-Tek, Norcross, GA, USA) according to the manufacturer's instructions. Negative controls were included during DNA extraction to check for potential contamination. DNA quality was assessed via 1% agarose gel electrophoresis, and DNA purity was determined using a NanoDrop 2000 spectrophotometer (Thermo Fisher Scientific, USA). Bacterial communities were characterized by amplifying the 16S rRNA V3–V4 region using the primers 799F (5′-AACMGGATTAGATACCCKG-3′) and 1193R (5′-ACGTCATCCCCACCTTCC-3′). Fungal communities were targeted using the ITS1F (5′-CTTGGTCATTTAGAGGAAGTAA-3′) and ITS2R (5′-GCTGCGTTCTTCATCGATGC-3′) primer pair, which amplifies the ITS region. PCR amplification was conducted in 25-μl reactions under the following conditions: initial denaturation at 95°C for 3 min, followed by 30 cycles of denaturation at 95°C for 30 s, annealing at 55°C for 30 s, and extension at 72°C for 45 s, with a final extension at 72°C for 10 min. Amplicons were verified by 2% agarose gel electrophoresis, purified using the AxyPrep DNA Gel Extraction Kit (Axygen Biosciences, USA), and quantified with the QuantiFluo™-ST system (Promega, USA). Sequencing libraries were constructed and sequenced on the Illumina MiSeq platform (Illumina, USA) using a 2 × 300 bp paired-end configuration. Raw sequence data were processed using the DADA2 plugin to perform quality filtering, denoising, merging, and chimera removal, resulting in ASV tables for downstream analysis. The taxonomic classification of the ASVs was performed using the Qiime2 classifier, with the Silva 16S rRNA database for bacterial communities and the UNITE fungal database for fungal communities to assign taxonomy at the phylum and genus levels [55].

### Rhizosphere metagenomic sequencing and analysis

Rhizosphere soil DNA from three composite samples per treatment was used to construct metagenomic libraries. Total DNA was sheared into ~350 bp fragments using a Covaris M220 ultrasonicator (Covaris, USA). Library preparation was performed using the NEBNext® Ultra™ II DNA Library Prep Kit (New England Biolabs, USA), which involved end-repair, A-tailing, adapter ligation, and PCR enrichment. Library quality was assessed with the Agilent 2100 Bioanalyzer (Agilent Technologies, USA), and concentrations were quantified using a Qubit 3.0 fluorometer (Invitrogen, USA). Qualified libraries were sequenced on the Illumina NovaSeq 6000 platform (Illumina, USA) using a 2 × 150 bp paired-end reads. Raw reads were subjected to quality control using fastp (v0.20.0) to remove adapter sequences, low-quality reads, and short fragments. High-quality reads were assembled using MEGAHIT (v1.2.9), followed by assembly optimization with SPAdes (v3.13.0). Gene prediction was performed using Prodigal (v2.6.3) with default settings to identify open reading frames (ORFs). To obtain a non-redundant unigene set, redundant sequences were removed using CD-HIT (v4.8.1) at a threshold of 95% sequence identity and 90% coverage. Gene abundance was estimated with Salmon (v1.6.0), and transcript abundance was expressed as TPM (transcripts per million). Functional annotation of unigenes was conducted using DIAMOND (v2.0.4) to align sequences against the NCBI NR, eggNOG, and KEGG databases (*E*-value <1^−5^). Special emphasis was placed on key genes involved in C and N cycling, such as *rbcL*, *abfA*, *napB*, *napC*, *nasA*, *nirK*, and *nirS* [50].

### Data visualization and statistical analysis

Alpha diversity indices (Chao1 and Shannon) for rhizosphere bacterial and fungal communities were calculated using the QIIME pipeline (v1.9.1), a widely used software for microbial community analysis. Principal coordinates analysis (PCoA) was conducted based on Bray-Curtis distance. One-way analysis of variance (ANOVA) was performed in SPSS 27.0, and treatment means were compared using Tukey's honestly significant difference test (*P* < 0.05). These analyses were employed to evaluate differences in plant growth, root traits, C and N content, and microbial community diversity and composition across different scion and N treatments. Spearman's correlation analysis was used to examine relationships among microbial community structure, plant traits, and soil C/N characteristics. Redundancy analysis (RDA) was employed to evaluate the influence of soil physicochemical properties on the relative abundance of dominant bacterial and fungal genera, as well as on functional genes associated with C and N cycling. The co-occurrence networks of rhizosphere microbes were constructed at the genus level using Spearman’s rank correlation method. We calculated the correlation matrix between microbial genera, and correlations with an absolute value of |r| > 0.6 were considered significant. To control for false positives, multiple-testing correction was applied using the Benjamini–Hochberg method. For each treatment group, which included three biological replicates, an individual network was constructed. The networks were generated using the SpiecEasi R package, and the network topological characteristics (node number, link number, average degree, and density) were visualized using Gephi software. Mantel tests were applied to assess correlations between microbial community diversity indices and plant/soil variables. Additionally, random forest modeling was used to evaluate the relative importance of rhizosphere microbial features and functional genes in predicting ^13^C and ^15^N uptake. Data visualization and figure generation were performed using Origin 2024, Canoco 5, Gephi, and R.

## Supplementary Material

Web_Material_uhaf334

## Data Availability

All relevant information and data are provided in the article and its supplementary materials.

## References

[1] Jin S, Li WJ, Cao YY. et al. Identifying barriers to sustainable apple production: a stakeholder perspective. J Environ Manag. 2022;302:114082 10.1016/j.jenvman.2021.114082 PMC8683745 34775335

[2] Luo C, Wang R, Li C. et al. Photosynthetic characteristics, soil nutrients, and their interspecific competitions in an apple–soybean alley cropping system subjected to different drip fertilizer regimes on the Loess Plateau, China. Agric Water Manag. 2023;275:108001

[3] Shah IH, Jinhui W, Li X. et al. Exploring the role of nitrogen and potassium in photosynthesis implications for sugar: accumulation and translocation in horticultural crops. Sci Hortic. 2024;327:112832

[4] Feng M, Augstein F, Kareem A. et al. Plant grafting: molecular mechanisms and applications. Mol Plant. 2024;17:75–91 38102831 10.1016/j.molp.2023.12.006

[5] Wang X, Xiong M, Xu J. et al. *PIN1a*-mediated auxin release from rootstock cotyledon contributes to healing in watermelon as revealed by seeds soaking-VIGS and cotyledon grafting. Hortic Res. 2024;12:uhae329 40051577 10.1093/hr/uhae329 PMC11883227

[6] Rodriguez-Izquierdo A, Carrasco D, Valledor L. et al. The scion-driven transcriptomic changes guide the resilience of grafted near-isohydric grapevines under water deficit. Hortic Res. 2024;12:uhae291 39906169 10.1093/hr/uhae291 PMC11789524

[7] Kuai J, Nie XY, Lou HX. et al. Nitrogen supply alleviates seed yield reduction by improving the morphology and carbon metabolism of pod walls in shaded rapeseed. Physiol Plantarum. 2023;175:e14003 10.1111/ppl.14003 37882291

[8] Xu X, Zhang X, Ni W. et al. Nitrogen-potassium balance improves leaf photosynthetic capacity by regulating leaf nitrogen allocation in apple. Hortic Res. 2023;11:uhad253 38486813 10.1093/hr/uhad253 PMC10939330

[9] Lou Y, Han Y, Miao Y. et al. A study of the soil water potential threshold values to trigger irrigation of ‘Shimizu Hakuto’ peach at pivotal fruit developmental stages. Hortic Plant J. 2024;10:376–86

[10] Wu L, Wu Y, Meng Y. et al. Environmental preferences of soil microbial attributes for long-term nitrogen and acid addition: from phylotype to community. Soil Biol Biochem. 2024;197:109541

[11] Ata-Ul-Karim ST, Cang L, Wang Y. et al. Effects of soil properties, nitrogen application, plant phenology, and their interactions on plant uptake of cadmium in wheat. J Hazard Mater. 2020;384:121452 31676167 10.1016/j.jhazmat.2019.121452

[12] Qiu L, Gou X, Kong Y. et al. Nitrogen addition stimulates N_2_O emissions via changes in denitrification community composition in a subtropical nitrogen-rich forest. J Environ Manag. 2023;348:119274 10.1016/j.jenvman.2023.119274 37890399

[13] Kelly C, Haddix ML, Byrne PF. et al. Divergent belowground carbon allocation patterns of winter wheat shape rhizosphere microbial communities and nitrogen cycling activities. Soil Biol Biochem. 2022;165:108518

[14] Rüger L, Feng K, Chen Y. et al. Responses of root architecture and the rhizosphere microbiome assembly of maize (*Zea mays* L.) to a soil texture gradient. Soil Biol Biochem. 2023;181:109026

[15] Li M, Wang J, Li N. et al. The rhizosphere contributes disproportionately to free-living nitrogen fixation in subalpine forest soils. Soil Biol Biochem. 2025;200:109641

[16] Chai X, Pi Y, Wang X. et al. Apple scion cultivars regulate root-rhizobacteria crosstalk through photosynthetic product-mediated sugar metabolism. Plant Cell Environ. 2025;48:6444–57 40375577 10.1111/pce.15614

[17] Chen L, Bian L, Ma Q. et al. Defensive alteration of root exudate composition by grafting *Prunus* sp. onto resistant rootstock contributes to reducing crown gall disease. Hortic Res. 2024;11:uhae049 38645683 10.1093/hr/uhae049 PMC11031412

[18] Zhang H, Yao D, Zhang G. et al. Effect of grafted scion varieties on apple root growth, carbon and nitrogen metabolism and microbiome in roots and rhizosphere soil. Appl Soil Ecol. 2025;206:105841

[19] Zhang J, Zhang WX, Ding CJ. et al. Non-additive gene expression in carbon and nitrogen metabolism drives growth heterosis in *Populus deltoides*. Plant Cell Environ. 2025;48:3529–43 39789702 10.1111/pce.15371 PMC11963483

[20] Kosola KR, Eller MS, Dohleman FG. et al. Short-stature and tall maize hybrids have a similar yield response to split-rate vs. pre-plant N applications, but differ in biomass and nitrogen partitioning. Field Crop Res. 2023;295:108880

[21] Liu Z, Ren H, Zhang S. et al. Effects of long-term nitrogen addition on root nitrogen acquisition strategy: insights from a 19-year experiment in two temperate tree species. For Ecol Manag. 2024;570:122220

[22] Pausch J, Holz M, Zhu B. et al. Rhizosphere priming promotes plant nitrogen acquisition by microbial necromass recycling. Plant Cell Environ. 2024;47:1987–96 38369964 10.1111/pce.14858

[23] Karkanis A, Ntatsi G, Vasilakakou E. et al. Combining *Tenebrio molitor* frass with inorganic nitrogen fertilizer to improve soil properties, growth parameters, and nutrient content of *Sonchus oleraceus* crop. Bioresour Technol. 2025;418:131901 39622418 10.1016/j.biortech.2024.131901

[24] Qiang B, Chen S, Fan Z. et al. Effects of nitrogen application levels on soybean photosynthetic performance and yield: insights from canopy nitrogen allocation studies. Field Crop Res. 2025;326:109871

[25] Wang YZ, Zhang YP, Yang ZY. et al. Intercropping improves maize yield and nitrogen uptake by regulating nitrogen transformation and functional microbial abundance in rhizosphere soil. J Environ Manag. 2024;358:120886 10.1016/j.jenvman.2024.120886 38648726

[26] Ghotbi M, Ghotbi M, Kuzyakov Y. et al. Management and rhizosphere microbial associations modulate genetic-driven nitrogen fate. Agric Ecosyst Environ. 2025;378:109308

[27] Séneca J, Söllinger A, Herbold CW. et al. Increased microbial expression of organic nitrogen cycling genes in long-term warmed grassland soils. ISME Commun. 2021;1:69 36759732 10.1038/s43705-021-00073-5 PMC9723740

[28] Zheng WW, Chun IJ, Hong SB. et al. Vegetative growth, mineral change, and fruit quality of ‘Fuji’ tree as affected by foliar seawater application. Agric Water Manag. 2013;126:97–103

[29] Marques de Carvalho L, de Oliveira Lopes Melo E, da Silva Filho FS. et al. Nitrogen rates-influence on proline and total nitrogen accumulation and fruit yield of young ‘Pera’ sweet orange on three rootstocks grown under rainfed condition. Sci Hortic. 2024;338:113755

[30] Cao Y, Du P, Li Z. et al. Melatonin promotes the recovery of apple plants after waterlogging by shaping the structure and function of the rhizosphere microbiome. Plant Cell Environ. 2024;47:2614–30 38712467 10.1111/pce.14903

[31] Hu M, Sardans J, Sun D. et al. Microbial diversity and keystone species drive soil nutrient cycling and multifunctionality following mangrove restoration. Environ Res. 2024;251:118715 38490631 10.1016/j.envres.2024.118715

[32] Li KX, Lin HM, Han M. et al. Soil metagenomics reveals the effect of nitrogen on soil microbial communities and nitrogen-cycle functional genes in the rhizosphere of *Panax ginseng*. Front Plant Sci. 2024;15:1411073 39170784 10.3389/fpls.2024.1411073 PMC11335670

[33] Lian J, Li G, Zhang J. et al. Nitrogen fertilization affected microbial carbon use efficiency and microbial resource limitations via root exudates. Sci Total Environ. 2024;950:174933 39043302 10.1016/j.scitotenv.2024.174933

[34] Marasco R, Alturkey H, Fusi M. et al. Rootstock–scion combination contributes to shape diversity and composition of microbial communities associated with grapevine root system. Environ Microbiol. 2022;24:3791–808 35581159 10.1111/1462-2920.16042 PMC9544687

[35] Wei X, Cui Y, Wang J. et al. Influence of scion cultivar on the rhizosphere microbiome and root exudates of *Phaseolus vulgaris* in grafting system. Plant Soil. 2024;503:415–32

[36] Chai X, Wang X, Li H. et al. Apple scion cultivars regulate the rhizosphere microbiota of scion/rootstock combinations. Appl Soil Ecol. 2022;170:104305

[37] Chai YN, Ge YF, Stoerger V. et al. High-resolution phenotyping of sorghum genotypic and phenotypic responses to low nitrogen and synthetic microbial communities. Plant Cell Environ. 2021;44:1611–26 33495990 10.1111/pce.14004

[38] Coskun D, Britto DT, Shi W. et al. How plant root exudates shape the nitrogen cycle. Trends Plant Sci. 2017;22:661–73 28601419 10.1016/j.tplants.2017.05.004

[39] Ning Q, Chen L, Zhang C. et al. Saprotrophic fungal communities in arable soils are strongly associated with soil fertility and stoichiometry. Appl Soil Ecol. 2021;159:103843

[40] Yan X, Guo S, Gao K. et al. The impact of the soil survival of the pathogen of *Fusarium* wilt on soil nutrient cycling mediated by microorganisms. Microorganisms. 2023;11:2207 37764051 10.3390/microorganisms11092207 PMC10537625

[41] Cui J, Zhu R, Wang X. et al. Effect of high soil C/N ratio and nitrogen limitation caused by the long-term combined organic-inorganic fertilization on the soil microbial community structure and its dominated SOC decomposition. J Environ Manag. 2022;303:114155 10.1016/j.jenvman.2021.114155 34861507

[42] Huang X, Yao K, Yu J. et al. Nitrogen removal performance and microbial characteristics during simultaneous chemical phosphorus removal process using Fe^3+^. Bioresour Technol. 2022;363:127972 36122847 10.1016/j.biortech.2022.127972

[43] Zhang C, Lei SL, Wu HY. et al. Simplified microbial network reduced microbial structure stability and soil functionality in alpine grassland along a natural aridity gradient. Soil Biol Biochem. 2024;191:109366

[44] Crutchfield-Peters KL, Rempe DM, Tune AK. et al. Linked nitrogen and carbon dynamics reveal distinct pools and patterns in a deep, weathered bedrock rhizosphere. Proc Natl Acad Sci USA. 2025;122:e2400452122 40343996 10.1073/pnas.2400452122 PMC12087964

[45] Whalen ED, Grandy AS, Geyer KM. et al. Microbial trait multifunctionality drives soil organic matter formation potential. Nat Commun. 2024;15:10209 39587087 10.1038/s41467-024-53947-2 PMC11589708

[46] Barrett J, Naduthodi MIS, Mao Y. et al. A promiscuous mechanism to phase separate eukaryotic carbon fixation in the green lineage. Nat Plants. 2024;10:1801–13 39384944 10.1038/s41477-024-01812-x PMC11570498

[47] Liao J, Dou Y, Yang X. et al. Soil microbial community and their functional genes during grassland restoration. J Environ Manag. 2023;325:116488 10.1016/j.jenvman.2022.116488 36419280

[48] Chang J, Costa OYA, Sun Y. et al. Domesticated rice alters the rhizosphere microbiome, reducing nitrogen fixation and increasing nitrous oxide emissions. Nat Commun. 2025;16:2038 40016229 10.1038/s41467-025-57213-x PMC11868393

[49] Xie ZH, Yu ZH, Li YS. et al. Soil microbial metabolism on carbon and nitrogen transformation links the crop-residue contribution to soil organic carbon. NPJ Biofilms Microbiomes. 2022;8:14 35365687 10.1038/s41522-022-00277-0 PMC8975862

[50] Gong Y, Hou R, Fu Q. et al. Modified biochar reduces the greenhouse gas emission intensity and enhances the net ecosystem economic budget in black soil soybean fields. Soil Till Res. 2024;237:105978

[51] Liao L, Wang J, Dijkstra FA. et al. Nitrogen enrichment stimulates rhizosphere multi-element cycling genes via mediating plant biomass and root exudates. Soil Biol Biochem. 2024;190:109306

[52] Tegtmeier R, Švara A, Gritsenko D. et al. *Malus sieversii*: a historical, genetic, and conservational perspective of the primary progenitor species of domesticated apples. Hortic Res. 2024;12:uhae244 39802738 10.1093/hr/uhae244 PMC11718403

[53] Xia H, Riaz M, Liu B. et al. Peanut shell biochar in acidic soil increases nitrogen absorption and photosynthesis characteristics of maize under different nitrogen levels. Environ Dev Sustain. 2023;25:8957–74

[54] Guan SY. Soil Enzymes and their Research Methods. Beijing: Agricultural Publishing House; 1986:

[55] Callahan BJ, McMurdie PJ, Rosen MJ. et al. DADA2: high-resolution sample inference from Illumina amplicon data. Nat Methods. 2016;13:581–3 27214047 10.1038/nmeth.3869 PMC4927377

