# Supplementary material for: Scion varieties and nitrogen levels affect carbon and nitrogen assimilation in apple via modulating rhizosphere microbial structure and function
Source: Hortic Res. 2025 Dec 3;13(3):uhaf334. doi: 10.1093/hr/uhaf334 (PMC13255517; doi:10.1093/hr/uhaf334)
Supplement: Web_Material_uhaf334 [file Web_Material_uhaf334.zip › Supplementary Material-Figure.docx]

**Scion varieties and nitrogen levels affect carbon and nitrogen assimilation in apple via modulating rhizosphere microbial structure and function**

**Running title:** Scion–nitrogen impact on apple rhizosphere microbiome

Huanhuan Zhang^1,2,3^, Wen Zhang^3^, Dongdong Yao^1,2,3^, Xujiao Li^1,2^, Hossam Salah Mahmoud Ali^1,2^, Jingshan Xi^1,2^, Yingchi Liang^1,2^, Fengyun Zhao^1,2,*^, Songlin Yu^1,2^, Kun Yu^1,2,*^

^1^ Department of Horticulture, College of Agriculture, Shihezi University, Shihezi 832003, China

^2^ Key Laboratory of Special Fruits and Vegetables Cultivation Physiology and Germplasm Resources Utilization of Xinjiang Production and Construction Corps, Department of Horticulture, College of Agriculture, Shihezi University, Shihezi 832003, China

^3^ Institute of Fruits and Vegetables, Xinjiang Academy of Agricultural Sciences, Urumqi 830091, China

***Corresponding authors:** Fengyun Zhao (zhaofengyun@shzu.edu.cn); Kun Yu (yukun@shzu.edu.cn)

Email addresses of authors:

Huanhuan Zhang: zhanghuanhuan@stu.shzu.edu.cn

Wen Zhang: zhangwenyys@xaas.ac.cn

Dongdong Yao: yaodongdong@stu.shzu.edu.cn

Xujiao Li: lixujiao@stu.shzu.edu.cn

Hossam Salah Mahmoud Ali: hossamsalah@mu.edu.eg

Jingshan Xi: xijingshan@stu.shzu.edu.cn

Yingchi Liang: liangyingchi@stu.shzu.edu.cn

Fengyun Zhao: zhaofengyun@shzu.edu.cn

Songlin Yu: songlin8900@sina.com

Kun Yu: yukun@shzu.edu.cn


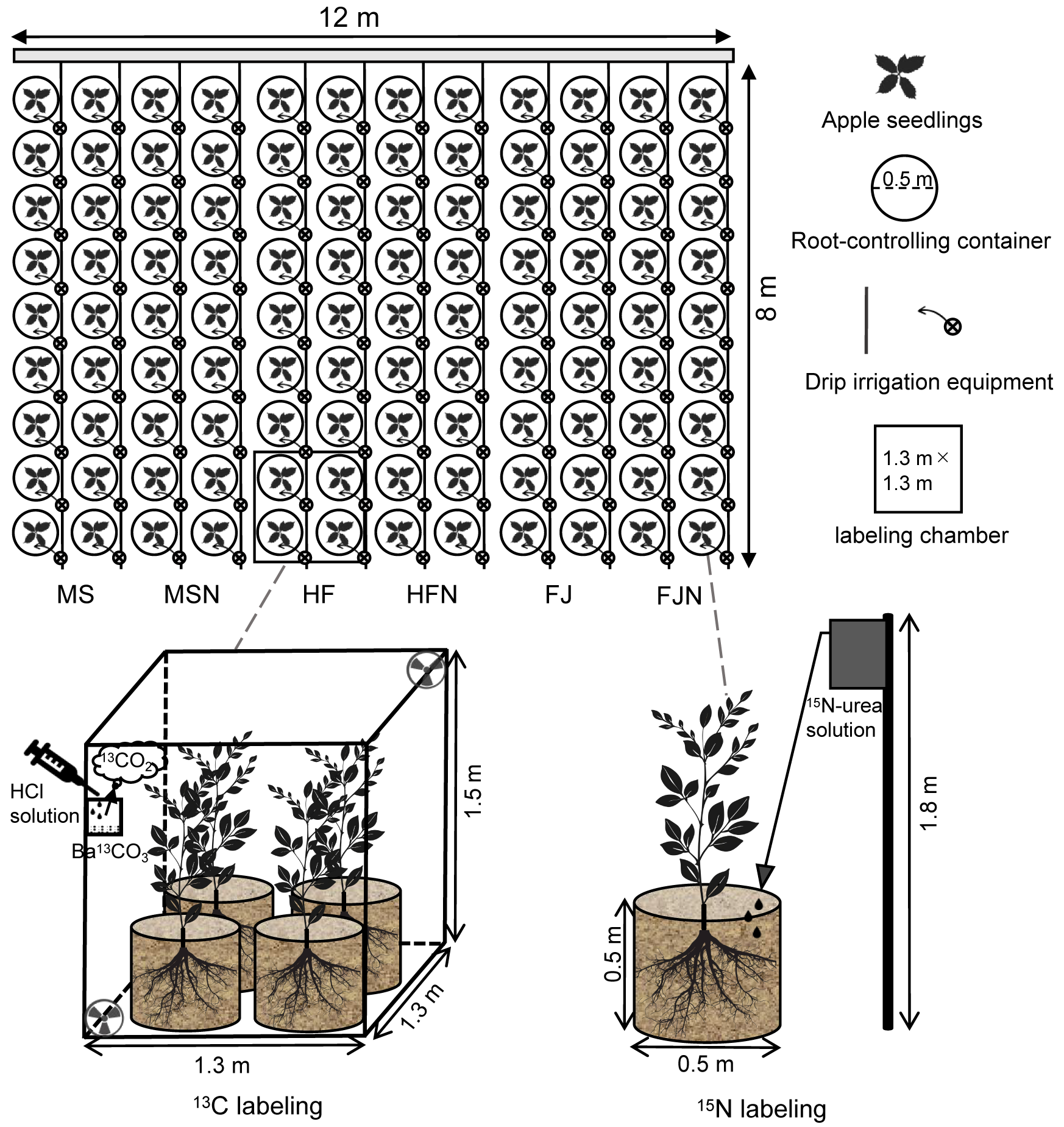


**Figure S1.** Design of the ^13^C and ^15^N double isotope labeling experiment. There are three different apple scion varieties and two nitrogen treatments in the study area. MS, *M. sieversii* grafted onto *M. sieversii*; HF, Hanfu grafted onto *M. sieversii*; FJ, Red Fuji grafted onto *M. sieversii.* N represents nitrogen application treatment.


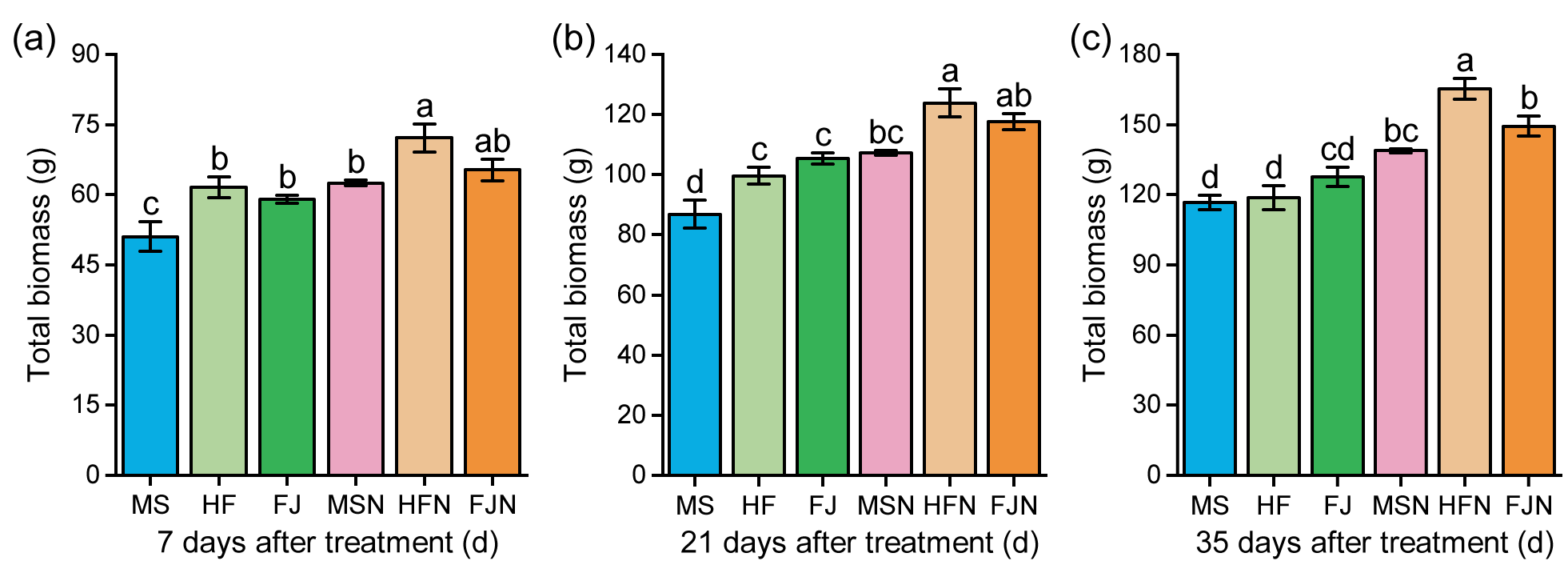


**Figure S2.** The total biomass of apple plants under different scion varieties and nitrogen levels. Values represent means ± SD (n = 3). Different lowercase letters above bars indicate significant differences among treatments within the same period based on Tukey’s honestly significant difference test (*P* < 0.05). MS, *M. sieversii* grafted onto *M. sieversii*; HF, Hanfu grafted onto *M. sieversii*; FJ, Red Fuji grafted onto *M. sieversii.* N represents nitrogen application treatment.

**
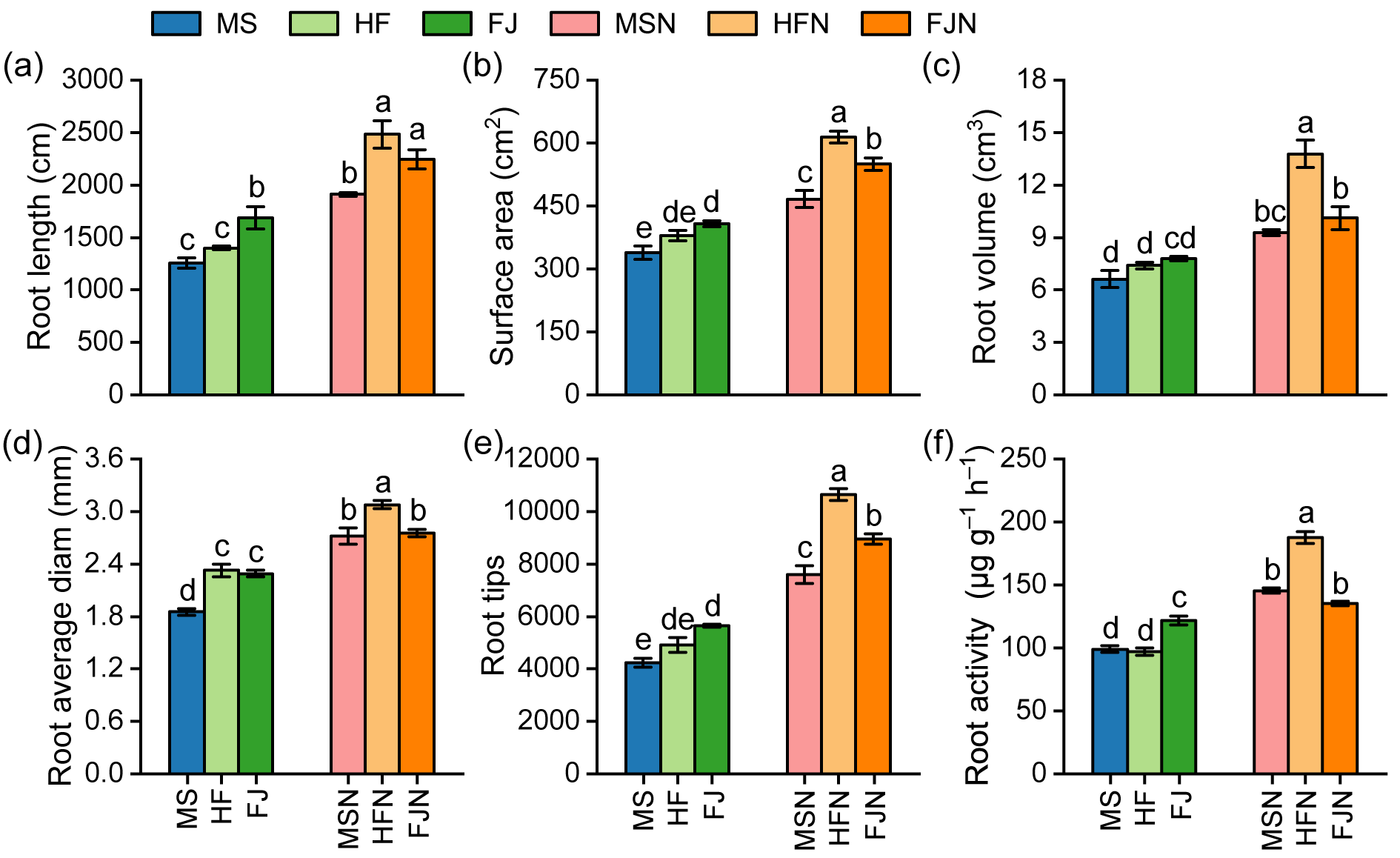
**

**Figure S3.** The (a) root length, (b) root surface area, (c) root volume, (d) root average diameter, (e) root tips, and (f) root activity of apple plants under different scion varieties and nitrogen levels. Values represent means ± SD (n = 3). Different lowercase letters above bars indicate significant differences among treatments based on Tukey’s honestly significant difference test (*P* < 0.05). MS, *M. sieversii* grafted onto *M. sieversii*; HF, Hanfu grafted onto *M. sieversii*; FJ, Red Fuji grafted onto *M. sieversii.* N represents nitrogen application treatment.


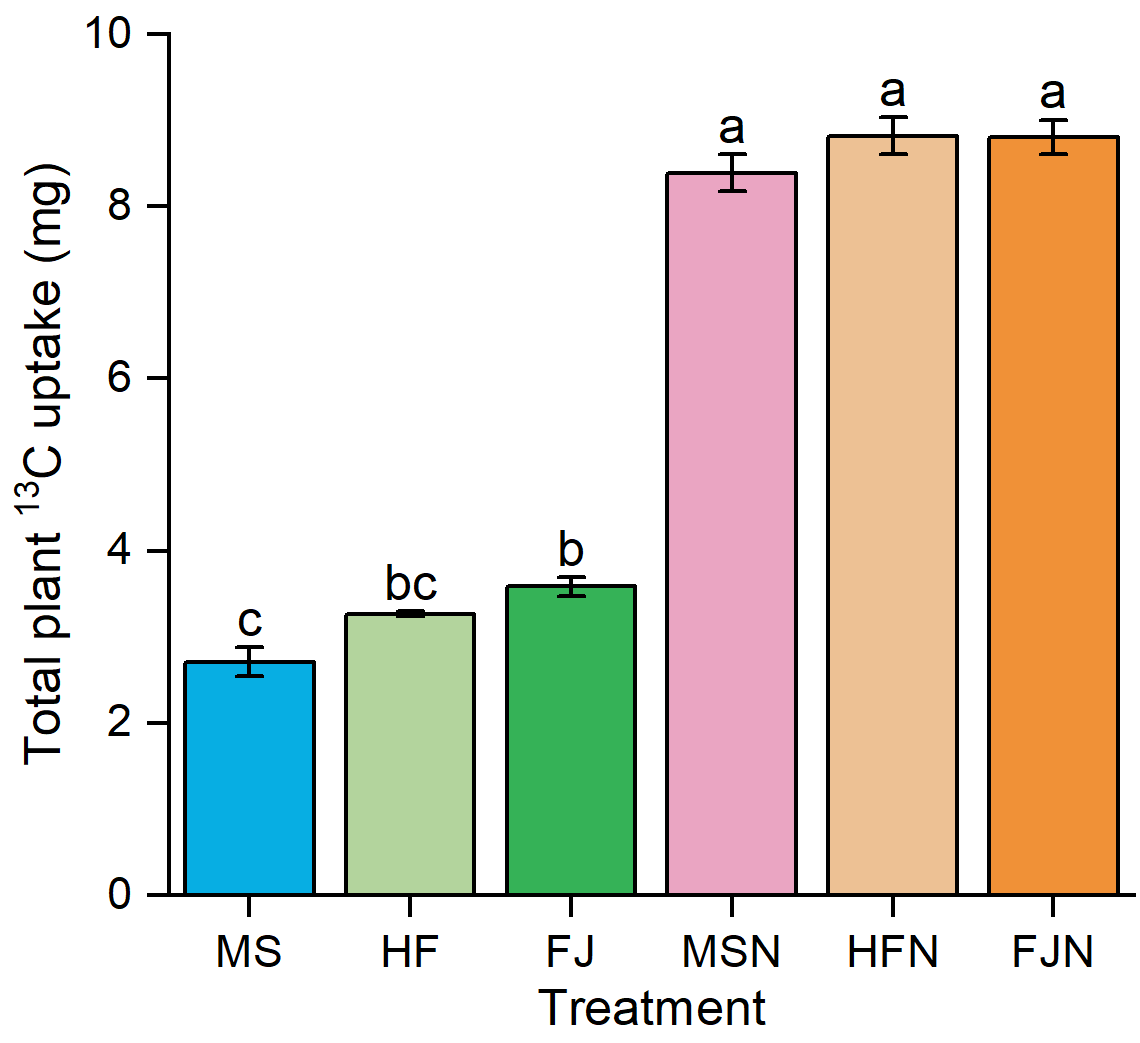


**Figure S4.** The total plant ^13^C uptake in apple under different scion varieties and nitrogen levels. Values represent means ± SD (n = 3). Different lowercase letters above bars indicate significant differences among treatments based on Tukey’s honestly significant difference test (*P* < 0.05). MS, *M. sieversii* grafted onto *M. sieversii*; HF, Hanfu grafted onto *M. sieversii*; FJ, Red Fuji grafted onto *M. sieversii.* N represents nitrogen application treatment.


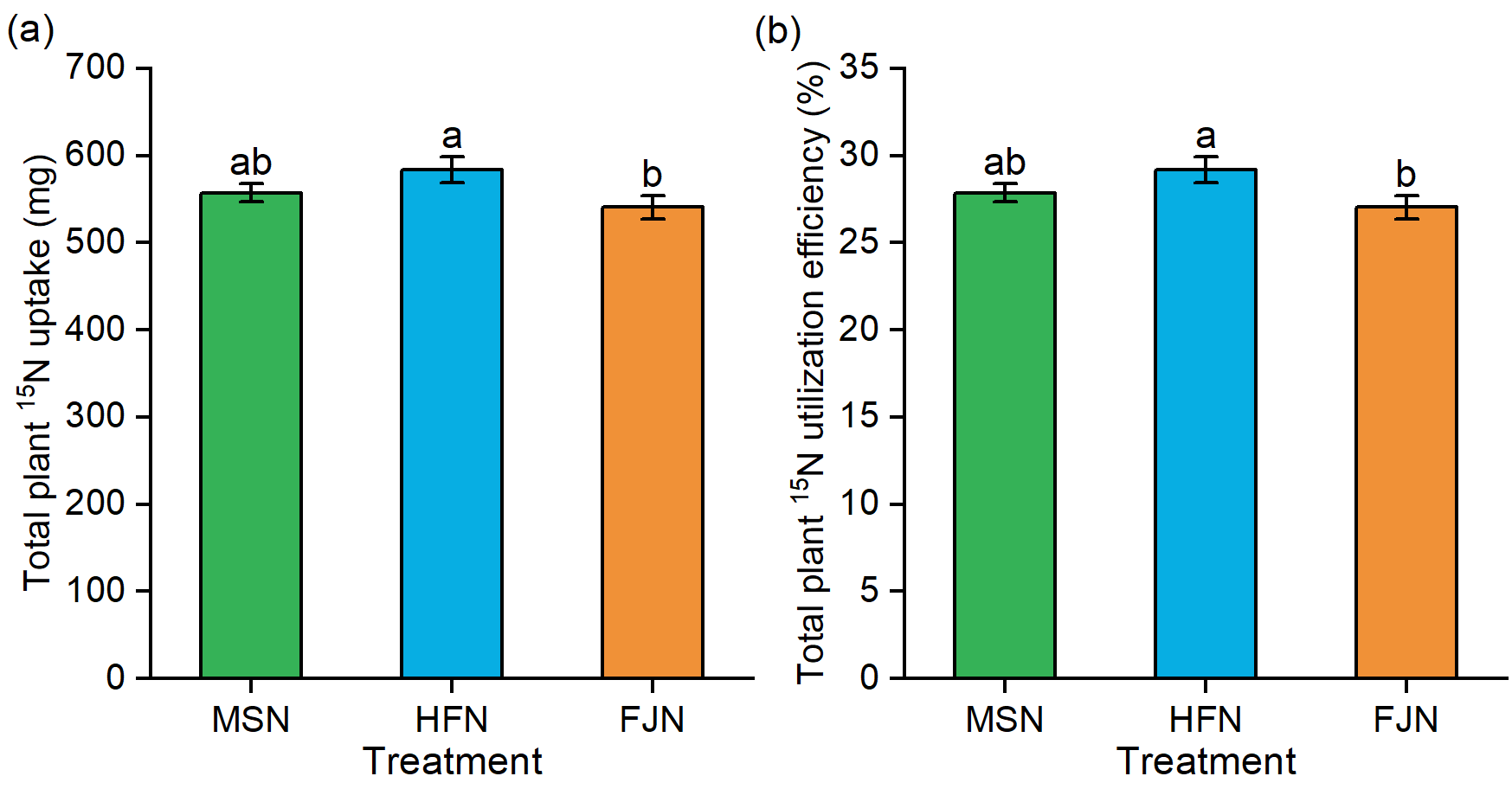


**Figure S5.** The total plant ^15^N (a) uptake and (b) utilization effciency in apple under different scion varieties and nitrogen levels. Values represent means ± SD (n = 3). Different lowercase letters above bars indicate significant differences among treatments based on Tukey’s honestly significant difference test (*P* < 0.05). MS, *M. sieversii* grafted onto *M. sieversii*; HF, Hanfu grafted onto *M. sieversii*; FJ, Red Fuji grafted onto *M. sieversii.* N represents nitrogen application treatment.

**~~
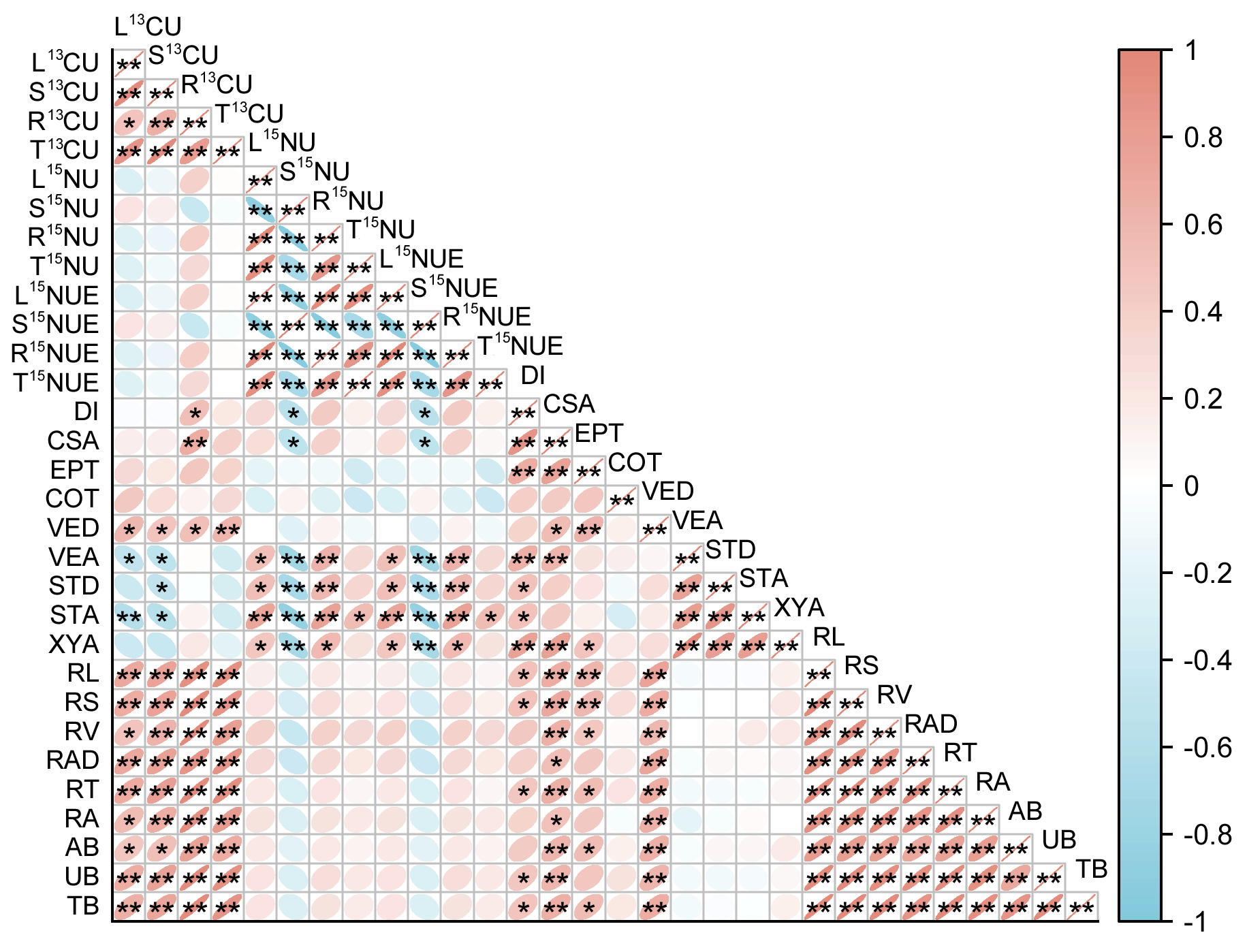
~~**

**Figure S6.** Correlations of plant ^13^C and ^15^N uptake with root tip anatomical characteristics and root and plant growth. Pairwise comparisons of factors are shown in the rectangle, with a colour gradient denoting Pearson's correlation coefficient. **P* < 0.05, ***P* < 0.01. L^13^CU, leaf ^13^C uptake; S^13^CU, stem ^13^C uptake; R^13^CU, root ^13^C uptake; T^13^CU, total plant ^13^C uptake; L^15^NU, leaf ^15^N uptake; S^15^NU, stem ^15^N uptake; R^15^NU, root ^15^N uptake; T^15^NU, total plant ^15^N uptake; L^15^NUE, leaf ^15^N utilization efficiency; S^15^NUE, stem ^15^N utilization efficiency; R^15^NUE, root ^15^N utilization efficiency; T^15^NUE, total plant ^15^N utilization efficiency; DI, root tip diameter; CSA, cross sectional area; EPT, epidermis thickness; COT, cortex thickness; VED, vessel diameter; VEA, vessel area; STD, stele diameter; STA, stele area; XYA, xylem area; RL, root length; RS, root surface area; RV, root volume; RAD, root average diameter; RT, root tips; RA, root activity; AB, aboveground biomass; UB, underground biomass; TB, total biomass.


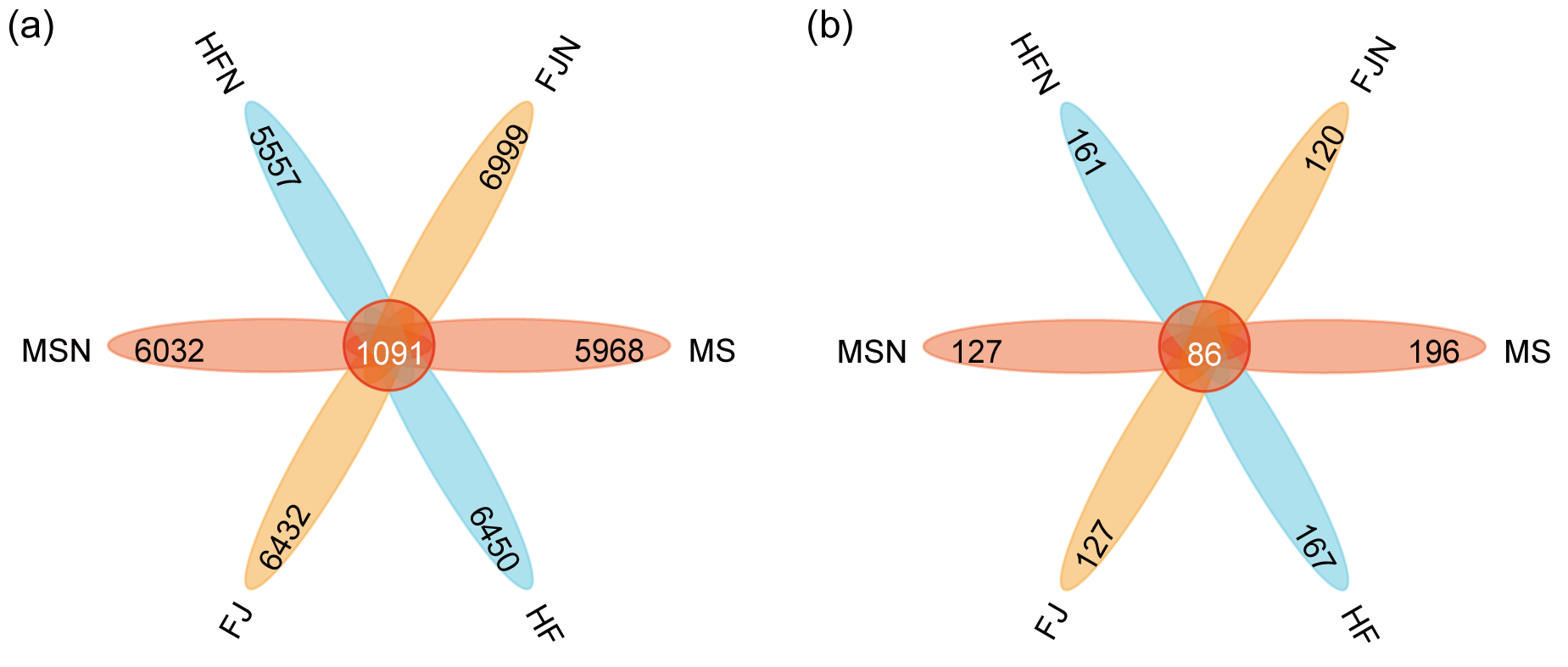


**Figure S7.** Venn diagram of soil (a) bacteria and (b) fungi in rhizosphere (the numbers in the Fig. represent the number of ASVs). MS, *M. sieversii* grafted onto *M. sieversii*; HF, Hanfu grafted onto *M. sieversii*; FJ, Red Fuji grafted onto *M. sieversii.* N represents nitrogen application treatment.


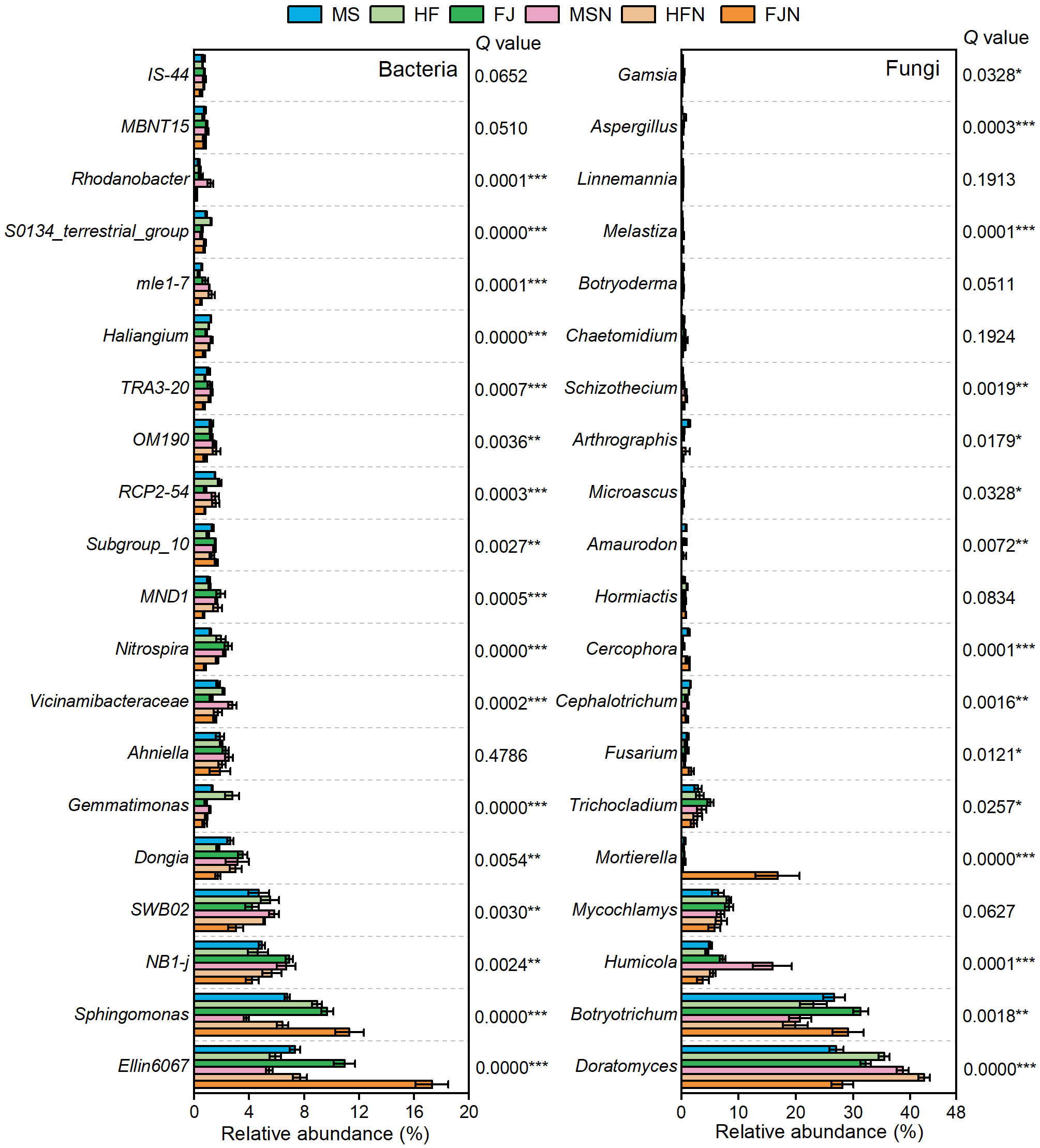


**Figure S8.** Relative abundance of the top 20 bacterial and fungal genera in rhizosphere samples from grafted apple plants with different scions under nitrogen treatment. The data are presented as the means ± standard deviations (n = 3). The different lowercase letters above the columns indicate significant differences between the scion treatments at the *P* < 0.05 level according to Tukey’s HSD test. MS, *M. sieversii* grafted onto *M. sieversii*; HF, Hanfu grafted onto *M. sieversii*; FJ, Red Fuji grafted onto *M. sieversii.* N represents nitrogen application treatment.


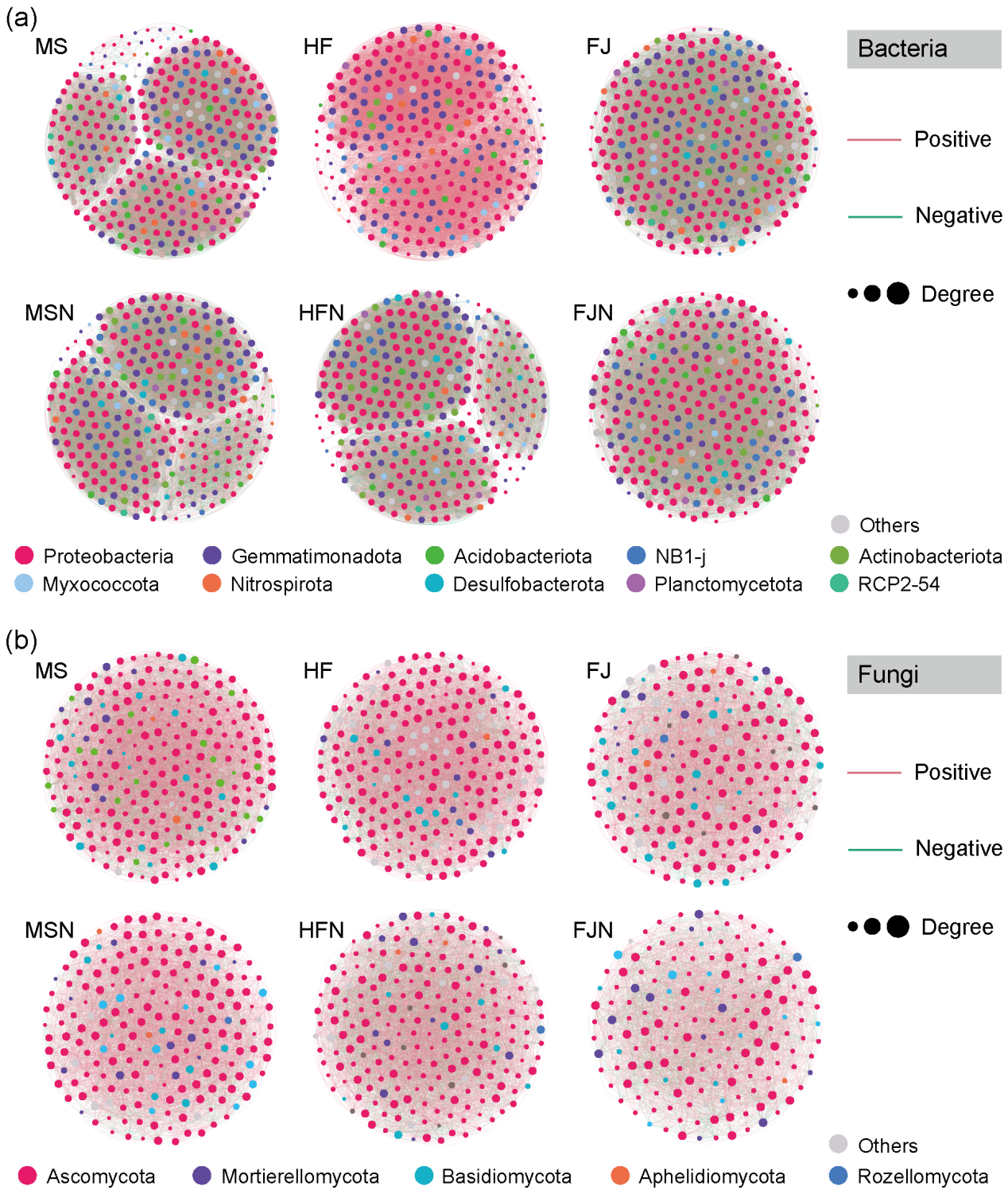


**Figure S9.** Co-occurrence networks of rhizosphere (a) bacterial and (b) fungal communities under different scion varieties and nitrogen levels. Node colors indicate taxonomic affiliation (phylum level), while node size reflects degree centrality (number of connections). MS, *M. sieversii* grafted onto *M. sieversii*; HF, Hanfu grafted onto *M. sieversii*; FJ, Red Fuji grafted onto *M. sieversii.* N represents nitrogen application treatment.


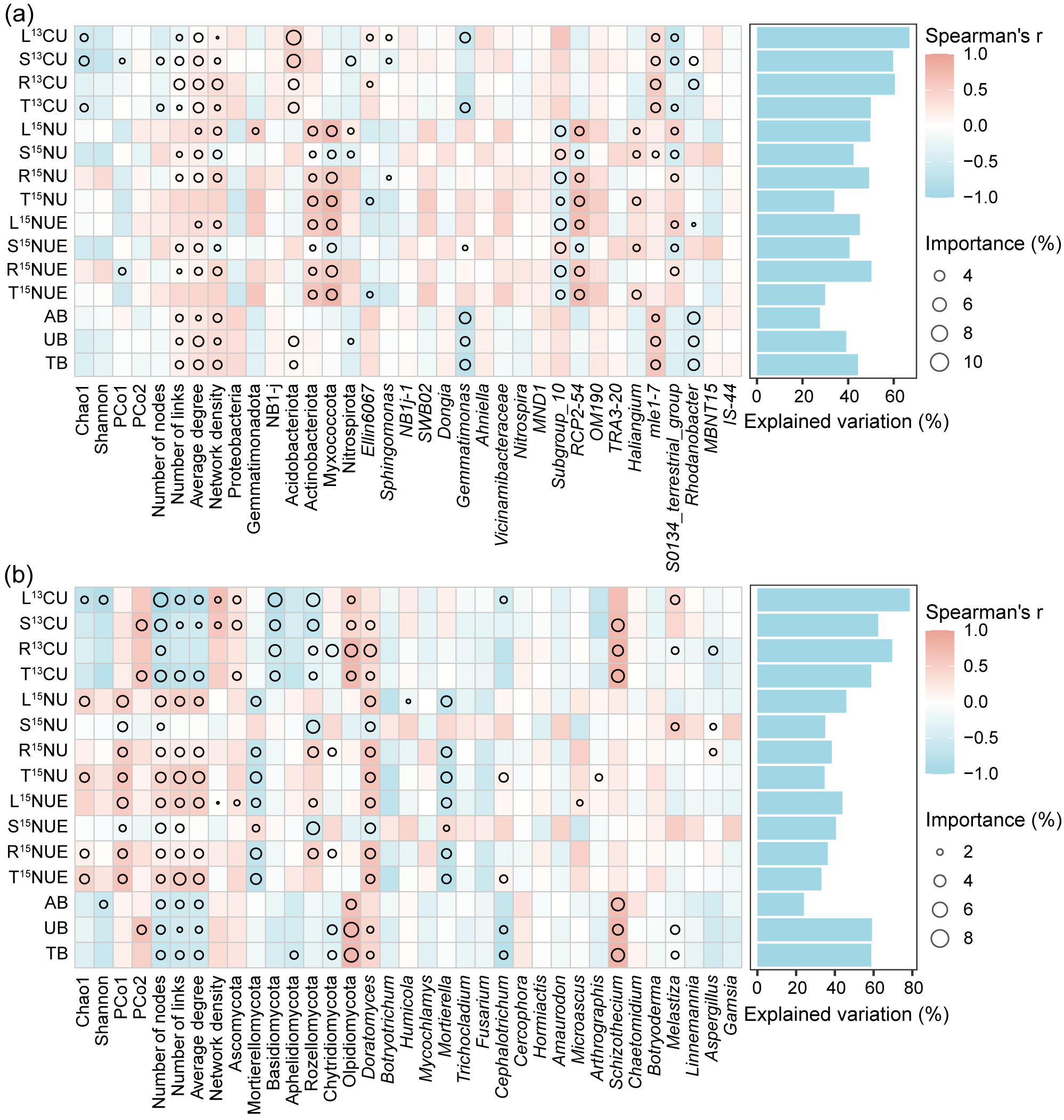


**Figure S10.** Random forest model results revealing the importance of rhizosphere (a) bacterial and (b) fungal community on plant carbon and nitrogen uptake. L^13^CU, leaf ^13^C uptake; S^13^CU, stem ^13^C uptake; R^13^CU, root ^13^C uptake; T^13^CU, total plant ^13^C uptake; L^15^NU, leaf ^15^N uptake; S^15^NU, stem ^15^N uptake; R^15^NU, root ^15^N uptake; T^15^NU, total plant ^15^N uptake; L^15^NUE, leaf ^15^N utilization efficiency; S^15^NUE, stem ^15^N utilization efficiency; R^15^NUE, root ^15^N utilization efficiency; T^15^NUE, total plant ^15^N utilization efficiency; AB, aboveground biomass; UB, underground biomass; TB, total biomass.

**
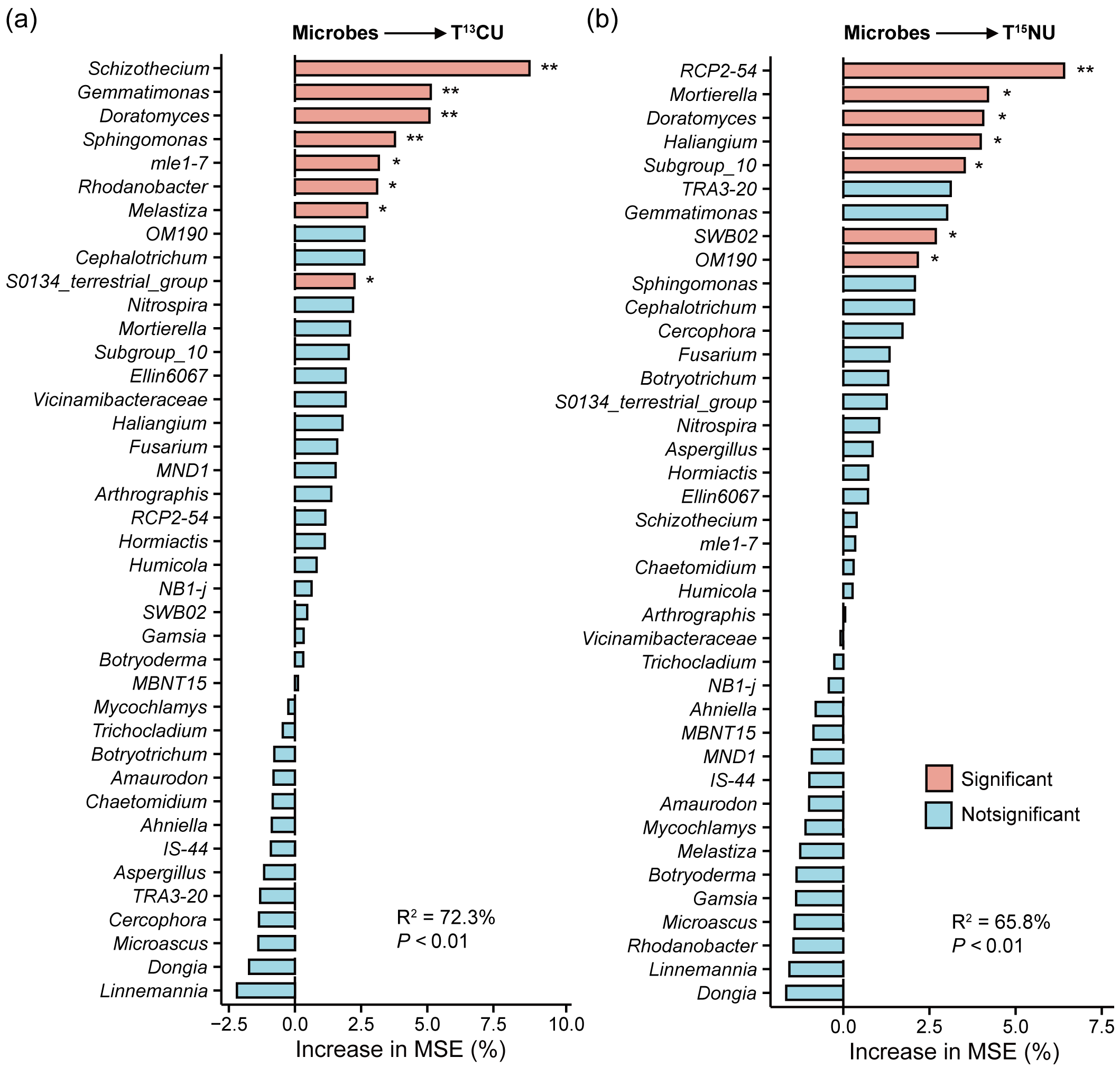
**

**Figure S11.** Random forest model results revealing the importance of rhizosphere bacteria and fungi at genus level on total plant (a) ^13^C and (b) ^15^N uptake. T^13^CU, total plant ^13^C uptake; T^15^NU, total plant ^15^N uptake. The significance levels are labelled as: *, *P* < 0.05; **, *P* < 0.01, respectively.


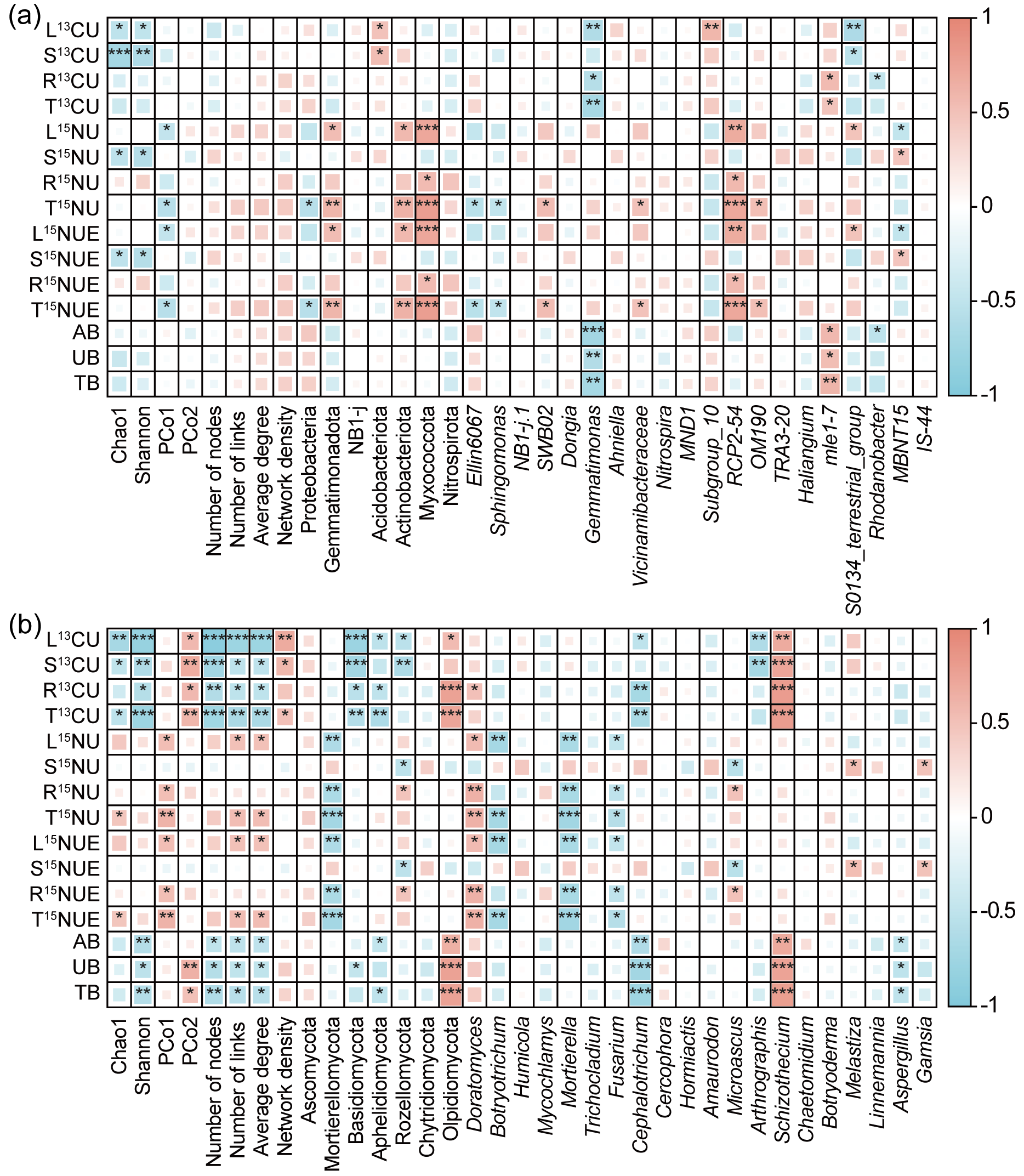


**Figure S12.** Spearman's correlation coefficients between rhizosphere (a) bacterial and (b) fungal community and carbon and nitrogen uptake. Pairwise comparisons of factors are shown in the rectangle, with a colour gradient denoting Spearman's correlation coefficient. **P* < 0.05, ***P* < 0.01, ****P* < 0.001. L^13^CU, leaf ^13^C uptake; S^13^CU, stem ^13^C uptake; R^13^CU, root ^13^C uptake; T^13^CU, total plant ^13^C uptake; L^15^NU, leaf ^15^N uptake; S^15^NU, stem ^15^N uptake; R^15^NU, root ^15^N uptake; T^15^NU, total plant ^15^N uptake; L^15^NUE, leaf ^15^N utilization efficiency; S^15^NUE, stem ^15^N utilization efficiency; R^15^NUE, root ^15^N utilization efficiency; T^15^NUE, total plant ^15^N utilization efficiency; AB, aboveground biomass; UB, underground biomass; TB, total biomass.

**
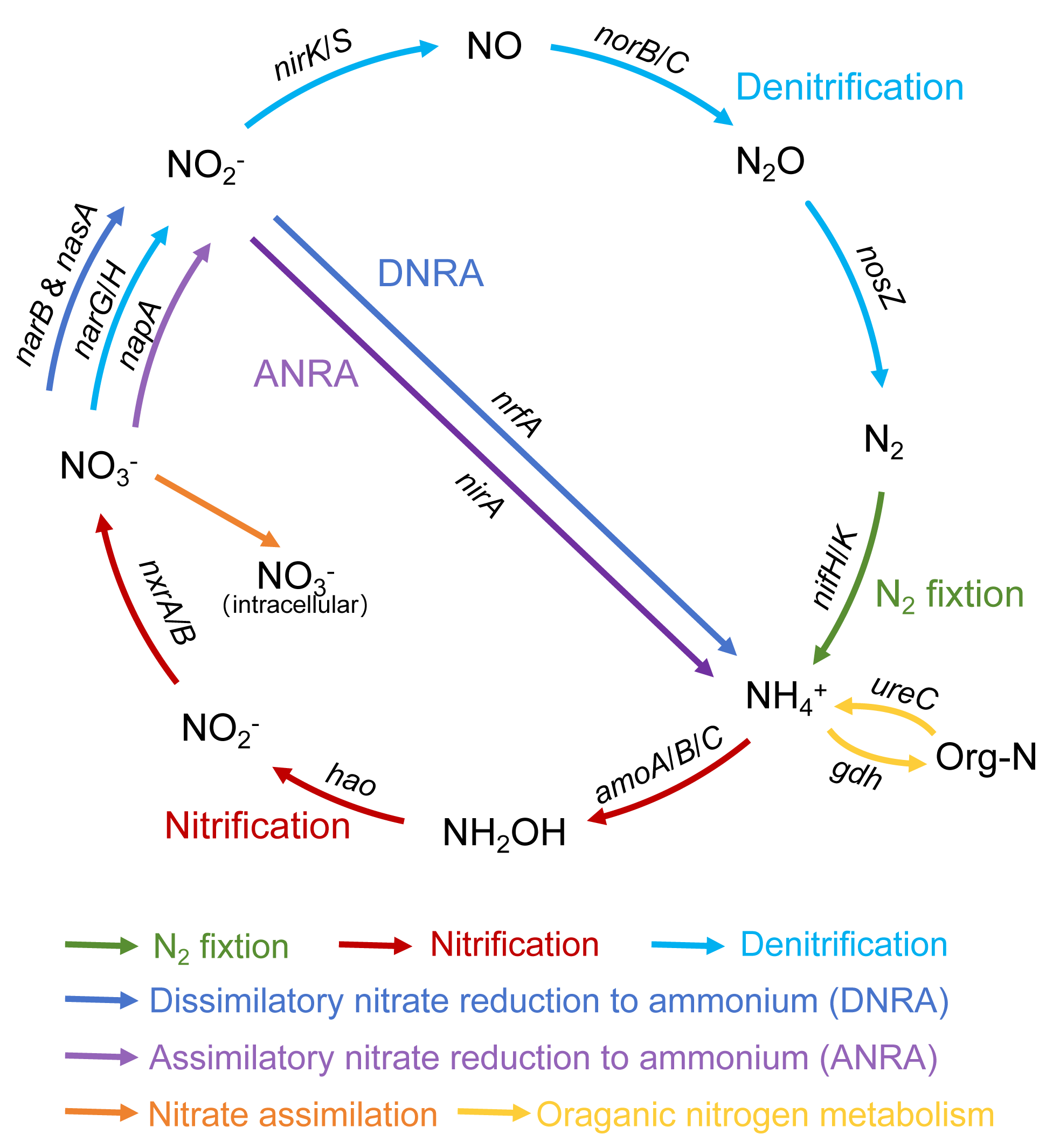
**

**Figure S13.** Functional genes and pathways involved in the nitrogen cycling.

**
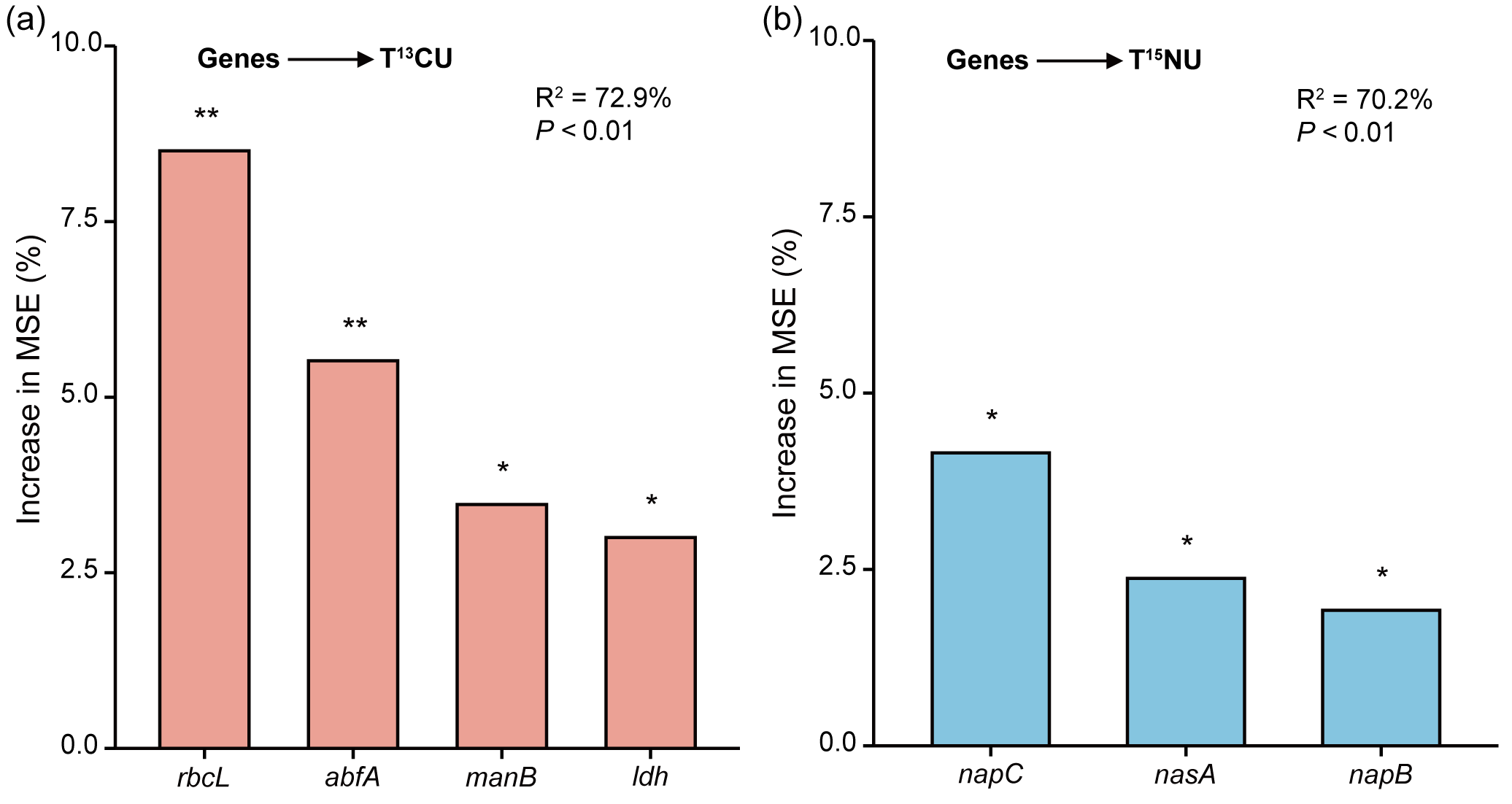
**

**Figure S14.** Random forest model results revealing the importance of rhizosphere (a) carbon and (b) nitrogen cycle functional genes on total plant ^13^C and ^15^N uptake, respectively. T^13^CU, total plant ^13^C uptake; T^15^NU, total plant ^15^N uptake. The significance levels are labelled as: *, *P* < 0.05; **, *P* < 0.01, respectively.


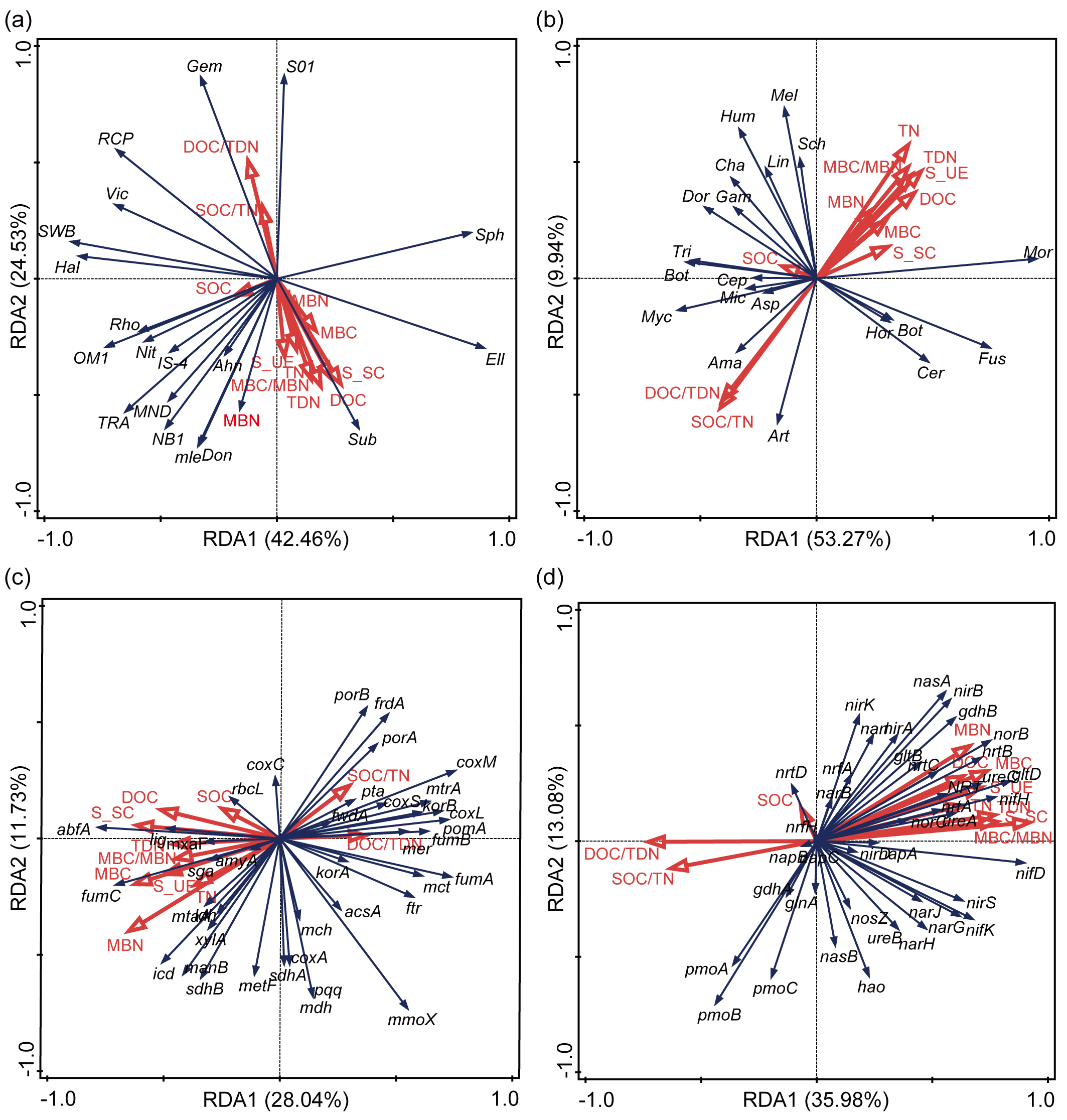


**Figure S15.** Redundancy analysis of rhizosphere soil physicochemical properties indices and dominant (a) bacterial and (b) fungal genera, and functional genes of (c) carbon and (d) nitrogen cycles. Dominant bacterial and fungal genera, and carbon and nitrogen cycle genes are indicated by blue vectors, and soil physicochemical variables are represented by red vectors. The positions and lengths of the arrows indicate the directions and strengths, respectively, of the effects of variables on microbial communities. Abbreviations: SOM, soil organic matter; TN, total nitrogen; DOC, dissolved organic carbon; TDN, total dissolved nitrogen; MBC, microbial biomass carbon; MBN, microbial biomass nitrogen; S_SC, soil sucrase; S_UE, soil urease; *Ell*, *Ellin6067*; *Sph*, *Sphingomonas*; *NB1*, *NB1-j*; *SWB*, *SWB02*; *Don*, *Dongia*; *Gem*, *Gemmatimonas*; *Ahn*, *Ahniella*; *Vic*, *Vicinamibacteraceae*; *Nit*, *Nitrospira*; *MND*, *MND1*; *Sub*, *Subgroup_10*; *RCP*, *RCP2-54*; *OM*1, *OM190*; *TRA*, *TRA3-20*; *Hal*, *Haliangium*; *mle*, *mle1-7*; *S01*, *S0134_terrestrial_group*; *Rho*, *Rhodanobacter*; *MBN*, *MBNT15*; *IS-4*, *IS-44*; *Dor*, *Doratomyces*; *Bot*, *Botryotrichum*; *Hum*, *Humicola*; *Myc*, *Mycochlamys*; *Mor*, *Mortierella*; *Tr*i, *Trichocladium*; *Fus*, *Fusarium*; *Cep*, *Cephalotrichum*; *Cer*, *Cercophora*; *Hor*, *Hormiactis*; *Ama*, *Amaurodon*; *Mic*, *Microascus*; *Art*, *Arthrographis*; *Sch*, *Schizothecium*; *Ch*a, *Chaetomidium*; *Bot*, *Botryoderma*; *Mel*, *Melastiz*a; *Lin*, *Linnemannia*; *Asp*, *Aspergillus*; *Gam*, *Gamsia*; *pqq*, *pqq-mdh*; *pmoA*, *pmoA-amoA*; *pmoB*, *pmoB-amoB*; *pmoC*, *pmoC-amoC*; *narG*, *narG/nxrA*; *narH*, *narH/nxrB*.
