# Supplementary material for: Scion varieties and nitrogen levels affect carbon and nitrogen assimilation in apple via modulating rhizosphere microbial structure and function
Source: Hortic Res. 2025 Dec 3;13(3):uhaf334. doi: 10.1093/hr/uhaf334 (PMC13255517; doi:10.1093/hr/uhaf334)
Supplement: Web_Material_uhaf334 [file Web_Material_uhaf334.zip › Supplementary Material-Table.docx]

**Scion varieties and nitrogen levels affect carbon and nitrogen assimilation in apple via modulating rhizosphere microbial structure and function**

**Running title:** Scion–nitrogen impact on apple rhizosphere microbiome

Huanhuan Zhang^1,2,3^, Wen Zhang^3^, Dongdong Yao^1,2,3^, Xujiao Li^1,2^, Hossam Salah Mahmoud Ali^1,2^, Jingshan Xi^1,2^, Yingchi Liang^1,2^, Fengyun Zhao^1,2,*^, Songlin Yu^1,2^, Kun Yu^1,2,*^

^1^ Department of Horticulture, College of Agriculture, Shihezi University, Shihezi 832003, China

^2^ Key Laboratory of Special Fruits and Vegetables Cultivation Physiology and Germplasm Resources Utilization of Xinjiang Production and Construction Corps, Department of Horticulture, College of Agriculture, Shihezi University, Shihezi 832003, China

^3^ Institute of Fruits and Vegetables, Xinjiang Academy of Agricultural Sciences, Urumqi 830091, China

***Corresponding authors:** Fengyun Zhao (zhaofengyun@shzu.edu.cn); Kun Yu (yukun@shzu.edu.cn)

Email addresses of authors:

Huanhuan Zhang: zhanghuanhuan@stu.shzu.edu.cn

Wen Zhang: zhangwenyys@xaas.ac.cn

Dongdong Yao: yaodongdong@stu.shzu.edu.cn

Xujiao Li: lixujiao@stu.shzu.edu.cn

Hossam Salah Mahmoud Ali: hossamsalah@mu.edu.eg

Jingshan Xi: xijingshan@stu.shzu.edu.cn

Yingchi Liang: liangyingchi@stu.shzu.edu.cn

Fengyun Zhao: zhaofengyun@shzu.edu.cn

Songlin Yu: songlin8900@sina.com

Kun Yu: yukun@shzu.edu.cn

**Table S1****.** The root tip anatomical characteristics of apple plants under different scion varieties and nitrogen levels.

| Indices | MS | HF | FJ | MSN | HFN | FJN |
| --- | --- | --- | --- | --- | --- | --- |
| DI (μm) | 1028.63 ± 18.13c | 1123.63 ± 17.24ab | 1136.93 ± 22.25ab | 1082.37 ± 15.80bc | 1197.77 ± 54.66a | 1168.67 ± 11.56ab |
| CSA (mm^2^) | 83.65 ± 0.84f | 96.98 ± 0.42d | 101.58 ± 0.60c | 90.70 ± 0.41e | 114.84 ± 0.50a | 111.67 ± 0.72b |
| EPT (μm) | 13.03 ± 0.31b | 13.00 ± 0.37b | 14.63 ± 0.37a | 13.07 ± 0.37b | 14.43 ± 0.25a | 14.83 ± 0.34a |
| COT (μm) | 195.47 ± 3.49b | 202.27 ± 3.75b | 201.07 ± 8.27b | 197.93 ± 12.57b | 196.27 ± 5.13b | 236.20 ± 4.89a |
| VED (μm) | 40.17 ± 0.68ab | 37.67 ± 0.87b | 42.33 ± 2.26a | 32.50 ± 1.00c | 42.23 ± 0.29a | 41.67 ± 0.90a |
| VEA (mm^2^) | 8.25 ± 0.49d | 16.87 ± 0.49a | 14.19 ± 0.52b | 12.15 ± 0.15c | 14.59 ± 0.58b | 13.57 ± 0.32bc |
| STD (μm) | 562.97 ± 13.48b | 664.40 ± 9.60a | 654.63 ± 12.52a | 568.43 ± 14.79b | 633.13 ± 13.50a | 587.87 ± 14.01b |
| STA (mm^2^) | 22.84 ± 0.27e | 37.20 ± 0.48a | 34.17 ± 0.24b | 27.14 ± 0.29c | 36.55 ± 0.21a | 24.59 ± 0.47d |
| XYA (mm^2^) | 16.78 ± 0.19e | 33.05 ± 0.31a | 31.28 ± 0.14b | 24.54 ± 0.85d | 32.48 ± 0.29ab | 29.62 ± 0.26c |

Values are means of 3 replicates ± SD followed by different letters differ significantly (Turkey's honestly significant difference test, *P* < 0.05). MS, *M. sieversii* grafted onto *M. sieversii*; HF, Hanfu grafted onto *M. sieversii*; FJ, Red Fuji grafted onto *M. sieversii.* N represents nitrogen application treatment. Abbreviations: DI, root tip diameter; CSA, cross sectional area; EPT, epidermis thickness; COT, cortex thickness; VED, vessel diameter; VEA, vessel area; STD, stele diameter; STA, stele area; XYA, xylem area.

**Table S2.** *P*-values for the growth indices of apple plants under different scion varieties and nitrogen treatments at 7, 21, and 35 days after treatment.

| Days after treatment | Indices | Scion | Nitrogen | Scion × Nitrogen |
| --- | --- | --- | --- | --- |
| 7 days | Plant height (cm) | 0.0119* | 0.0000*** | 0.1021ns |
|  | Stem diameter (mm) | 0.3331ns | 0.0022** | 0.396ns |
|  | Aboveground biomass (g) | 0.0005*** | 0.0003*** | 0.2629ns |
|  | Underground biomass (g) | 0.0034** | 0.0000*** | 0.0021** |
|  | Total biomass (g) | 0.0001*** | 0.0000*** | 0.2597ns |
| 21 days | Plant height (cm) | 0.0001*** | 0.0000*** | 0.3213ns |
|  | Stem diameter (mm) | 0.0032** | 0.0005*** | 0.1454ns |
|  | Aboveground biomass (g) | 0.0003*** | 0.0019** | 0.0707ns |
|  | Underground biomass (g) | 0.0029** | 0.0000*** | 0.5817ns |
|  | Total biomass (g) | 0.0000*** | 0.0000*** | 0.0616ns |
| 35 days | Plant height (cm) | 0.0077** | 0.0000*** | 0.0517ns |
|  | Stem diameter (mm) | 0.3588ns | 0.0000*** | 0.573ns |
|  | Aboveground biomass (g) | 0.1177ns | 0.0016** | 0.0661ns |
|  | Underground biomass (g) | 0.0013** | 0.0000*** | 0.0069** |
|  | Total biomass (g) | 0.0006*** | 0.0000*** | 0.0009*** |

*, **, and *** indicate significant differences at *P* < 0.05, 0.01, and 0.001 levels, respectively; ns indicates no significant difference.

**Table S3.** *P*-values for physiological indices of apple plants under different scion varieties and nitrogen treatments.

| Indices | Scion | Nitrogen | Scion × Nitrogen |
| --- | --- | --- | --- |
| Root length (cm) | 0.0000*** | 0.0000*** | 0.0013** |
| Root surface area (cm^2^) | 0.0000*** | 0.0000*** | 0.0005*** |
| Root volume (cm^3^) | 0.0000*** | 0.0000*** | 0.0001*** |
| Root average diameter (mm) | 0.0000*** | 0.0000*** | 0.0012** |
| Root tips | 0.0000*** | 0.0000*** | 0.0000*** |
| Root activity (μg g^-1^ h^-1^) | 0.0000*** | 0.0000*** | 0.0000*** |
| Root diameter (μm) | 0.0002*** | 0.0056** | 0.5642ns |
| Cross sectional area (mm^2^) | 0.0000*** | 0.0000*** | 0.0000*** |
| Epidermis thickness (μm) | 0.0001*** | 0.0147* | 0.0246* |
| Cortex thickness (μm) | 0.0017** | 0.0246* | 0.0036** |
| Vessel diameter (μm) | 0.0001*** | 0.0878ns | 0.0000*** |
| Vessel area (mm^2^) | 0.0000*** | 0.217ns | 0.0000*** |
| Stele diameter (μm) | 0.0000*** | 0.0015** | 0.0073** |
| Stele area (mm^2^) | 0.0000*** | 0.0000*** | 0.0000*** |
| Xylem area (mm^2^) | 0.0000*** | 0.0000*** | 0.0000*** |
| Leaf ^13^C uptake (mg) | 0.0000*** | 0.0000*** | 0.0000*** |
| Stem ^13^C uptake (mg) | 0.0007*** | 0.0000*** | 0.0003*** |
| Root ^13^C uptake (mg) | 0.0000*** | 0.0000*** | 0.0000*** |
| Total plant ^13^C uptake (mg) | 0.0004*** | 0.0000*** | 0.1833ns |
| Leaf ^13^C distribution ratio (%) | 0.0000*** | 0.0000*** | 0.0000*** |
| Stem ^13^C distribution ratio (%) | 0.0108* | 0.0283* | 0.1311ns |
| Root ^13^C distribution ratio (%) | 0.0000*** | 0.0000*** | 0.0000*** |

*, **, and *** indicate significant differences at *P* < 0.05, 0.01, and 0.001 levels, respectively; ns indicates no significant difference.

**Table S4.** The number of sequences, ASVs counts, and Good’s coverage index in the rhizosphere bacteria of apple plants under different scion varieties and nitrogen levels.

| Sample ID | Sample type | Original sequences | High-quality sequences | ASVs counts |
| --- | --- | --- | --- | --- |
| Rhizosphere soil 1 | MS | 147,497 | 42,333 | 4,649 |
| Rhizosphere soil 2 | MS | 133,139 | 45,379 | 4,502 |
| Rhizosphere soil 3 | MS | 139,680 | 44,934 | 4,367 |
| Rhizosphere soil 4 | HF | 134,462 | 44,179 | 4,606 |
| Rhizosphere soil 5 | HF | 148,305 | 38,544 | 4,885 |
| Rhizosphere soil 6 | HF | 140,267 | 40,265 | 4,420 |
| Rhizosphere soil 7 | FJ | 134,853 | 41,124 | 4,487 |
| Rhizosphere soil 8 | FJ | 139,385 | 40,919 | 4,422 |
| Rhizosphere soil 9 | FJ | 141,060 | 43,547 | 4,825 |
| Rhizosphere soil 10 | MSN | 143,348 | 42,321 | 4,312 |
| Rhizosphere soil 11 | MSN | 140,304 | 45,350 | 4,432 |
| Rhizosphere soil 12 | MSN | 134,304 | 42,735 | 4,126 |
| Rhizosphere soil 13 | HFN | 137,984 | 42,211 | 4,342 |
| Rhizosphere soil 14 | HFN | 141,800 | 42,090 | 4,420 |
| Rhizosphere soil 15 | HFN | 135,825 | 39,600 | 4,126 |
| Rhizosphere soil 16 | FJN | 122,422 | 42,286 | 4,070 |
| Rhizosphere soil 17 | FJN | 139,711 | 46,689 | 4,544 |
| Rhizosphere soil 18 | FJN | 148,180 | 45,626 | 5,016 |

MS, *M. sieversii* grafted onto *M. sieversii*; HF, Hanfu grafted onto *M. sieversii*; FJ, Red Fuji grafted onto *M. sieversii.* N represents nitrogen application treatment.

**Table S5.** The number of sequences, ASVs counts, and Good’s coverage index in the rhizosphere fungi of apple plants under different scion varieties and nitrogen levels.

| Sample ID | Sample type | Original sequences | High-quality sequences | ASVs counts |
| --- | --- | --- | --- | --- |
| Rhizosphere soil 1 | MS | 144,590 | 125,714 | 212 |
| Rhizosphere soil 2 | MS | 147,823 | 127,539 | 250 |
| Rhizosphere soil 3 | MS | 135,219 | 114,770 | 233 |
| Rhizosphere soil 4 | HF | 139,200 | 121,366 | 181 |
| Rhizosphere soil 5 | HF | 146,340 | 127,696 | 231 |
| Rhizosphere soil 6 | HF | 147,827 | 124,375 | 202 |
| Rhizosphere soil 7 | FJ | 141,149 | 123,224 | 185 |
| Rhizosphere soil 8 | FJ | 135,486 | 116,587 | 198 |
| Rhizosphere soil 9 | FJ | 147,754 | 130,173 | 161 |
| Rhizosphere soil 10 | MSN | 136,215 | 119,114 | 171 |
| Rhizosphere soil 11 | MSN | 136,010 | 105,939 | 152 |
| Rhizosphere soil 12 | MSN | 129,147 | 110,166 | 185 |
| Rhizosphere soil 13 | HFN | 146,693 | 126,780 | 216 |
| Rhizosphere soil 14 | HFN | 144,005 | 125,289 | 212 |
| Rhizosphere soil 15 | HFN | 145,461 | 121,280 | 205 |
| Rhizosphere soil 16 | FJN | 115,582 | 100,786 | 174 |
| Rhizosphere soil 17 | FJN | 114,418 | 95,490 | 162 |
| Rhizosphere soil 18 | FJN | 118,945 | 103,333 | 169 |

MS, *M. sieversii* grafted onto *M. sieversii*; HF, Hanfu grafted onto *M. sieversii*; FJ, Red Fuji grafted onto *M. sieversii.* N represents nitrogen application treatment.

**Table S6.** Relative abundance of the top 5 bacterial and fungal phyla in rhizosphere of apple plants under different scion varieties and nitrogen levels.

| Microbiome | Dominant phylum | MS | HF | FJ | MSN | HFN | FJN |
| --- | --- | --- | --- | --- | --- | --- | --- |
| Bacterial phyla | Proteobacteria | 58.28 ± 1.12b | 52.14 ± 1.52c | 61.86 ± 0.77b | 52.94 ± 0.42c | 59.44 ± 1.40b | 67.38 ± 1.20a |
|  | Gemmatimonadota | 14.85 ± 0.57c | 21.82 ± 1.43a | 10.90 ± 0.59d | 18.36 ± 1.01b | 14.08 ± 1.01c | 10.90 ± 0.68d |
|  | NB1-j | 4.26 ± 0.42b | 5.31 ± 0.27ab | 6.92 ± 0.26a | 6.03 ± 0.48ab | 5.65 ± 0.70ab | 4.23 ± 1.07b |
|  | Acidobacteriota | 4.88 ± 0.25b | 5.06 ± 0.17ab | 4.46 ± 0.36b | 5.86 ± 0.22a | 4.45 ± 0.44b | 5.98 ± 0.19a |
|  | Actinobacteriota | 2.28 ± 0.05c | 2.94 ± 0.09b | 1.41 ± 0.15d | 3.63 ± 0.23a | 2.84 ± 0.18bc | 1.55 ± 0.29d |
| Fungal phyla | Ascomycota | 87.007 ± 0.989c | 89.268 ± 1.109bc | 90.155 ± 0.786bc | 95.182 ± 0.438a | 92.164 ± 0.213ab | 72.874 ± 1.560d |
|  | Mortierellomycota | 7.746 ± 0.635b | 1.922 ± 0.527c | 4.989 ± 0.425bc | 1.834 ± 0.230c | 1.558 ± 0.194c | 23.313 ± 2.548a |
|  | Basidiomycota | 2.587 ± 0.539a | 0.633 ± 0.037c | 1.932 ± 0.604ab | 0.477 ± 0.043c | 1.026 ± 0.159bc | 0.381 ± 0.134c |
|  | Aphelidiomycota | 0.071 ± 0.018a | 0.079 ± 0.029a | 0.061 ± 0.013a | 0.063 ± 0.014a | 0.082 ± 0.059a | 0.025 ± 0.012a |
|  | Rozellomycota | 0.015 ± 0.003bc | 0.071 ± 0.028a | 0.055 ± 0.009ab | 0.004 ± 0.001c | 0.038 ± 0.008abc | 0.004 ± 0.003c |

Values are means of 3 replicates ± SD followed by different letters differ significantly (Turkey's honestly significant difference test, *P* < 0.05). MS, *M. sieversii* grafted onto *M. sieversii*; HF, Hanfu grafted onto *M. sieversii*; FJ, Red Fuji grafted onto *M. sieversii.* N represents nitrogen application treatment.

**Table S****7.** Relative abundance of the top 20 bacterial genera in rhizosphere of grafted apple plants under different scion varieties and nitrogen levels.

| Bacterial genera | MS | HF | FJ | MSN | HFN | FJN |
| --- | --- | --- | --- | --- | --- | --- |
| *Ellin6067* | 7.33 ± 0.38cd | 5.89 ± 0.41cd | 10.94 ± 0.76b | 5.47 ± 0.24d | 7.70 ± 0.51c | 17.30 ± 1.18a |
| *Sphingomonas* | 6.77 ± 0.17c | 8.94 ± 0.35b | 9.68 ± 0.44ab | 3.80 ± 0.18d | 6.44 ± 0.40c | 11.31 ± 1.05a |
| *NB1-j* | 4.92 ± 0.23bc | 4.64 ± 0.74c | 6.92 ± 0.26a | 6.70 ± 0.69ab | 5.65 ± 0.70abc | 4.23 ± 0.46c |
| *SWB02* | 4.70 ± 0.74ab | 5.52 ± 0.67a | 4.21 ± 0.48ab | 5.82 ± 0.36a | 5.09 ± 0.05a | 3.03 ± 0.54b |
| *Dongia* | 2.62 ± 0.22ab | 1.72 ± 0.12b | 3.54 ± 0.34a | 3.14 ± 0.84ab | 3.02 ± 0.45ab | 1.72 ± 0.18b |
| *Gemmatimonas* | 1.31 ± 0.05b | 2.77 ± 0.51a | 0.83 ± 0.08b | 1.14 ± 0.07b | 0.87 ± 0.10b | 0.76 ± 0.16b |
| *Ahniella* | 1.87 ± 0.30a | 1.97 ± 0.11a | 2.30 ± 0.23a | 2.53 ± 0.28a | 2.03 ± 0.26a | 1.88 ± 0.76a |
| *Vicinamibacteraceae* | 1.73 ± 0.13bc | 2.15 ± 0.08ab | 1.24 ± 0.12c | 2.77 ± 0.30a | 1.72 ± 0.30bc | 1.49 ± 0.10bc |
| *Nitrospira* | 1.18 ± 0.05cd | 1.94 ± 0.33ab | 2.47 ± 0.26a | 2.19 ± 0.11ab | 1.67 ± 0.08bc | 0.77 ± 0.07d |
| *MND1* | 1.06 ± 0.11bc | 1.13 ± 0.07bc | 1.93 ± 0.32a | 1.60 ± 0.08ab | 1.71 ± 0.32ab | 0.68 ± 0.05c |
| *Subgroup_10* | 1.33 ± 0.08ab | 0.99 ± 0.03b | 1.52 ± 0.04a | 1.48 ± 0.11a | 1.29 ± 0.18ab | 1.60 ± 0.11a |
| *RCP2-54* | 1.52 ± 0.03a | 1.84 ± 0.02a | 0.80 ± 0.11b | 1.52 ± 0.26a | 1.57 ± 0.26a | 0.80 ± 0.04b |
| *OM190* | 1.24 ± 0.13ab | 1.20 ± 0.07ab | 1.26 ± 0.09ab | 1.49 ± 0.14a | 1.63 ± 0.27a | 0.79 ± 0.14b |
| *TRA3-20* | 1.04 ± 0.11ab | 0.79 ± 0.03bc | 1.15 ± 0.16a | 1.28 ± 0.06a | 1.12 ± 0.08a | 0.69 ± 0.07c |
| *Haliangium* | 1.22 ± 0.02ab | 1.06 ± 0.02bc | 0.88 ± 0.05cd | 1.27 ± 0.08a | 1.08 ± 0.04b | 0.72 ± 0.08d |
| *mle1-7* | 0.56 ± 0.04cd | 0.32 ± 0.07d | 0.80 ± 0.20bc | 1.09 ± 0.01ab | 1.27 ± 0.21a | 0.50 ± 0.06cd |
| *S0134_terrestrial_group* | 0.89 ± 0.05b | 1.23 ± 0.03a | 0.56 ± 0.05cd | 0.51 ± 0.07d | 0.78 ± 0.08b | 0.72 ± 0.06bc |
| *Rhodanobacter* | 0.32 ± 0.07b | 0.40 ± 0.07b | 0.47 ± 0.16b | 1.18 ± 0.21a | 0.17 ± 0.05b | 0.17 ± 0.03b |
| *MBNT15* | 0.78 ± 0.06a | 0.67 ± 0.06a | 0.91 ± 0.04a | 0.93 ± 0.10a | 0.75 ± 0.10a | 0.75 ± 0.09a |
| *IS-44* | 0.67 ± 0.11a | 0.61 ± 0.01a | 0.73 ± 0.05a | 0.73 ± 0.11a | 0.71 ± 0.03a | 0.49 ± 0.10a |

Values are means of 3 replicates ± SD followed by different letters differ significantly (Turkey's honestly significant difference test, *P* < 0.05). MS, *M. sieversii* grafted onto *M. sieversii*; HF, Hanfu grafted onto *M. sieversii*; FJ, Red Fuji grafted onto *M. sieversii.* N represents nitrogen application treatment.**Table S8.** Relative abundance of the top 20 fungal genera in rhizosphere of apple plants under different scion varieties and nitrogen levels.

| Fungal genera | MS | HF | FJ | MSN | HFN | FJN |
| --- | --- | --- | --- | --- | --- | --- |
| *Doratomyces* | 27.088 ± 1.207d | 35.428 ± 0.961bc | 32.219 ± 0.915c | 38.672 ± 1.060ab | 42.435 ± 0.980a | 28.107 ± 1.891d |
| *Botryotrichum* | 26.695 ± 1.894abc | 23.098 ± 2.358bc | 31.316 ± 1.303a | 20.768 ± 1.976c | 19.914 ± 2.164c | 29.105 ± 2.700ab |
| *Humicola* | 5.087 ± 0.289b | 4.474 ± 0.278b | 7.228 ± 0.495b | 15.904 ± 3.407a | 5.521 ± 0.511b | 3.772 ± 1.004b |
| *Mycochlamys* | 6.413 ± 1.053a | 8.308 ± 0.399a | 8.339 ± 0.686a | 6.848 ± 0.671a | 6.968 ± 0.988a | 5.775 ± 1.011a |
| *Mortierella* | 0.612 ± 0.152b | 0.406 ± 0.089b | 0.496 ± 0.040b | 0.551 ± 0.200b | 0.276 ± 0.062b | 16.807 ± 3.824a |
| *Trichocladium* | 2.947 ± 0.630ab | 3.231 ± 0.672ab | 5.095 ± 0.552a | 3.549 ± 0.828ab | 2.862 ± 0.746ab | 2.175 ± 0.518b |
| *Fusarium* | 1.128 ± 0.180ab | 0.798 ± 0.208b | 0.999 ± 0.325ab | 0.568 ± 0.201b | 0.559 ± 0.075b | 1.733 ± 0.430a |
| *Cephalotrichum* | 1.589 ± 0.032a | 1.346 ± 0.056ab | 0.885 ± 0.191bc | 1.180 ± 0.152ab | 0.680 ± 0.098c | 0.936 ± 0.234bc |
| *Cercophora* | 1.305 ± 0.171a | 0.252 ± 0.082b | 0.405 ± 0.152b | 0.067 ± 0.030b | 1.084 ± 0.350a | 1.423 ± 0.081a |
| *Hormiactis* | 0.455 ± 0.183a | 1.011 ± 0.114a | 0.565 ± 0.192a | 0.575 ± 0.281a | 0.503 ± 0.170a | 0.791 ± 0.088a |
| *Amaurodon* | 0.810 ± 0.082a | 0.019 ± 0.005b | 0.651 ± 0.250ab | 0.062 ± 0.028b | 0.419 ± 0.386ab | 0.027 ± 0.019b |
| *Microascus* | 0.096 ± 0.040b | 0.570 ± 0.082a | 0.284 ± 0.141ab | 0.264 ± 0.073ab | 0.299 ± 0.206ab | 0.147 ± 0.084b |
| *Arthrographis* | 1.369 ± 0.202a | 0.384 ± 0.164ab | 0.368 ± 0.065b | 0.000 ± 0.000ab | 0.761 ± 0.691ab | 0.275 ± 0.077b |
| *Schizothecium* | 0.274 ± 0.063c | 0.320 ± 0.042c | 0.448 ± 0.082bc | 0.710 ± 0.177ab | 0.897 ± 0.128a | 0.504 ± 0.098bc |
| *Chaetomidium* | 0.309 ± 0.238a | 0.348 ± 0.089a | 0.612 ± 0.101a | 0.756 ± 0.371a | 0.540 ± 0.237a | 0.229 ± 0.047a |
| *Botryoderma* | 0.344 ± 0.121a | 0.183 ± 0.087a | 0.289 ± 0.083a | 0.318 ± 0.115a | 0.349 ± 0.071a | 0.056 ± 0.027a |
| *Melastiza* | 0.176 ± 0.055b | 0.219 ± 0.039b | 0.268 ± 0.027ab | 0.461 ± 0.053a | 0.070 ± 0.023b | 0.223 ± 0.142b |
| *Linnemannia* | 0.251 ± 0.040a | 0.286 ± 0.084a | 0.318 ± 0.060a | 0.366 ± 0.040a | 0.257 ± 0.033a | 0.236 ± 0.034a |
| *Aspergillus* | 0.170 ± 0.053bc | 0.656 ± 0.138a | 0.362 ± 0.075b | 0.324 ± 0.046b | 0.067 ± 0.018c | 0.217 ± 0.051bc |
| *Gamsia* | 0.232 ± 0.054a | 0.213 ± 0.069a | 0.379 ± 0.144a | 0.387 ± 0.068a | 0.129 ± 0.085a | 0.115 ± 0.049a |

Values are means of 3 replicates ± SD followed by different letters differ significantly (Turkey's honestly significant difference test, *P* < 0.05). MS, *M. sieversii* grafted onto *M. sieversii*; HF, Hanfu grafted onto *M. sieversii*; FJ, Red Fuji grafted onto *M. sieversii.* N represents nitrogen application treatment.

**Table S9.** Topological properties of bacterial and fungal communities networks in rhizosphere of apple plants under different scion varieties and nitrogen levels.

| Microbiome | Treatment | Number of nodes | Number of links | Average degree | Network density | Positive (%) | Negative (%) |
| --- | --- | --- | --- | --- | --- | --- | --- |
| Bacteria | MS | 315 | 14,119 | 89.64 | 0.285 | 56.86 | 43.14 |
|  | HF | 277 | 11,326 | 81.78 | 0.296 | 100.00 | 0.00 |
|  | FJ | 280 | 11,983 | 85.59 | 0.307 | 50.25 | 49.75 |
|  | MSN | 295 | 13,331 | 90.38 | 0.307 | 50.00 | 50.00 |
|  | HFN | 296 | 14,145 | 95.57 | 0.324 | 50.32 | 49.68 |
|  | FJN | 264 | 9,529 | 72.19 | 0.274 | 50.33 | 49.67 |
| Fungi | MS | 245 | 4,935 | 40.29 | 0.165 | 73.43 | 26.57 |
|  | HF | 229 | 4,391 | 38.35 | 0.168 | 74.65 | 25.35 |
|  | FJ | 199 | 3,268 | 32.84 | 0.166 | 72.61 | 27.39 |
|  | MSN | 198 | 3,273 | 33.06 | 0.168 | 79.87 | 20.13 |
|  | HFN | 220 | 4,002 | 36.38 | 0.166 | 70.06 | 29.94 |
|  | FJN | 164 | 2,399 | 29.26 | 0.179 | 70.20 | 29.80 |

MS, *M. sieversii* grafted onto *M. sieversii*; HF, Hanfu grafted onto *M. sieversii*; FJ, Red Fuji grafted onto *M. sieversii.* N represents nitrogen application treatment.

**Table S10.** Physicochemical properties and enzyme activities of rhizosphere soil in apple plants under different scion varieties and nitrogen levels.

| Treatment | SOM (g kg^-1^) | TN (mg kg^-1^) | SOM/TN | DOC (mg kg^-1^) | TDN (mg kg^-1^) | DOC/TDN | MBC (mg kg^-1^) | MBN (mg kg^-1^) | MBC/MBN | S_SC  (mg g^-1^ d^-1^) | S_UE  (μg g^-1^ d^-1^) |
| --- | --- | --- | --- | --- | --- | --- | --- | --- | --- | --- | --- |
| MS | 18.00 ± 0.22a | 1.20 ± 0.01b | 15.06 ± 0.28a | 118.82 ± 5.29b | 328.26 ± 8.02d | 0.36 ± 0.01ab | 377.93 ± 6.53d | 45.53 ± 0.65d | 1044.78 ± 16.22c | 3.44 ± 0.07d | 211.51 ± 4.37c |
| HF | 17.98 ± 0.24a | 1.20 ± 0.00b | 14.99 ± 0.23a | 121.24 ± 3.98b | 315.40 ± 6.64d | 0.38 ± 0.01a | 392.03 ± 4.07cd | 52.04 ± 1.29bc | 1020.61 ± 32.17c | 3.50 ± 0.11d | 227.66 ± 3.65c |
| FJ | 18.09 ± 0.05a | 1.19 ± 0.01b | 15.19 ± 0.14a | 128.03 ± 4.47ab | 371.53 ± 9.54c | 0.34 ± 0.02b | 388.04 ± 3.89cd | 48.05 ± 1.22cd | 1128.15 ± 63.98c | 3.72 ± 0.13cd | 220.42 ± 5.81c |
| MSN | 18.06 ± 0.30a | 1.37 ± 0.02a | 13.15 ± 0.04b | 131.92 ± 3.67ab | 504.60 ± 12.20b | 0.26 ± 0.01c | 400.01 ± 3.25bc | 53.71 ± 2.65bc | 1530.32 ± 24.86b | 3.84 ± 0.08c | 336.44 ± 8.55b |
| HFN | 18.06 ± 0.33a | 1.39 ± 0.01a | 13.00 ± 0.26b | 138.85 ± 3.60a | 573.21 ± 10.80a | 0.24 ± 0.01c | 420.34 ± 5.03a | 60.07 ± 1.69a | 1737.10 ± 77.09a | 4.57 ± 0.10a | 378.60 ± 10.79a |
| FJN | 17.96 ± 0.29a | 1.38 ± 0.02a | 13.00 ± 0.32b | 138.90 ± 2.54a | 560.10 ± 13.06a | 0.25 ± 0.01c | 409.81 ± 4.14ab | 55.94 ± 2.05ab | 1653.13 ± 52.17ab | 4.18 ± 0.07b | 358.50 ± 2.98ab |

Values are means of 3 replicates ± SD followed by different letters differ significantly (Turkey's honestly significant difference test, *P* < 0.05). MS, *M. sieversii* grafted onto *M. sieversii*; HF, Hanfu grafted onto *M. sieversii*; FJ, Red Fuji grafted onto *M. sieversii.* N represents nitrogen application treatment. Abbreviations: SOM, soil organic matter; TN, total nitrogen; DOC, dissolved organic carbon; TDN, total dissolved nitrogen; MBC, microbial biomass carbon; MBN, microbial biomass nitrogen; S_SC, soil sucrase; S_UE, soil urease.

**Table S11.** Abundance of the carbon cycle functional genes in rhizosphere of apple plants under different scion varieties and nitrogen levels.

| Carbon cycle genes | MS | HF | FJ | MSN | HFN | FJN |
| --- | --- | --- | --- | --- | --- | --- |
| *acsA* | 221.05 ± 5.91a | 216.12 ± 2.94a | 211.8 ± 2.89a | 225.45 ± 4.62a | 221.94 ± 7.04a | 214.16 ± 10.94a |
| *rbcL* | 37.40 ± 1.94ab | 28.64 ± 1.06c | 34.50 ± 1.44bc | 38.37 ± 1.86ab | 41.20 ± 2.00a | 40.16 ± 1.99ab |
| *mct* | 128.97 ± 3.59a | 123.92 ± 5.79a | 127.41 ± 2.90a | 131.96 ± 4.08a | 124.45 ± 4.40a | 122.80 ± 2.55a |
| *frdA* | 97.75 ± 8.97ab | 76.34 ± 1.15b | 99.87 ± 9.93a | 85.14 ± 6.23ab | 80.76 ± 2.10ab | 84.69 ± 5.14ab |
| *korA* | 404.12 ± 8.69a | 410.78 ± 18.37a | 396.65 ± 19.36a | 404.17 ± 15.44a | 402.17 ± 13.85a | 410.49 ± 21.07a |
| *korB* | 265.43 ± 8.70a | 251.06 ± 7.13a | 255.28 ± 24.68a | 258.79 ± 4.98a | 249.43 ± 2.64a | 252.53 ± 6.25a |
| *mdh* | 281.03 ± 6.84ab | 300.53 ± 1.85a | 280.56 ± 6.44b | 287.88 ± 6.97ab | 279.93 ± 7.49b | 283.21 ± 3.43ab |
| *icd* | 385.20 ± 17.58ab | 416.35 ± 2.64a | 375.54 ± 12.63b | 390.36 ± 13.79ab | 419.27 ± 12.12a | 406.91 ± 3.14ab |
| *sdhA* | 560.88 ± 15.31ab | 605.47 ± 18.6a | 551.41 ± 18.03b | 587.26 ± 2.18ab | 581.17 ± 13.82ab | 575.79 ± 19.51ab |
| *sdhB* | 290.58 ± 7.38b | 322.95 ± 2.54a | 283.26 ± 10.47b | 304.35 ± 2.17ab | 315.17 ± 5.83a | 301.83 ± 8.02ab |
| *fumA* | 21.52 ± 4.80a | 17.18 ± 1.71a | 15.48 ± 2.46a | 18.54 ± 1.87a | 14.16 ± 1.13a | 16.64 ± 2.89a |
| *fumB* | 7.23 ± 2.96a | 3.17 ± 0.27a | 3.17 ± 1.12a | 5.20 ± 1.67a | 2.85 ± 0.94a | 2.91 ± 0.68a |
| *fumC* | 284.15 ± 1.72b | 302.44 ± 2.92ab | 296.51 ± 9.48ab | 297.97 ± 7.70ab | 311.78 ± 6.51a | 306.44 ± 0.95a |
| *metF* | 255.65 ± 5.21ab | 280.78 ± 5.80a | 253.54 ± 9.33b | 262.16 ± 11.85ab | 266.64 ± 5.28ab | 266.80 ± 5.72ab |
| *pta* | 69.84 ± 4.46a | 68.85 ± 7.74a | 73.21 ± 5.66a | 71.61 ± 7.13a | 66.38 ± 4.84a | 71.71 ± 4.38a |
| *amyA* | 26.82 ± 4.28a | 26.82 ± 3.27a | 28.16 ± 8.7a | 26.94 ± 1.49a | 32.90 ± 1.77a | 27.99 ± 2.30a |
| *sga* | 36.01 ± 5.69a | 36.69 ± 1.66a | 37.42 ± 2.93a | 40.08 ± 1.57a | 40.50 ± 3.50a | 39.94 ± 0.97a |
| *abfA* | 107.88 ± 4.55c | 123.27 ± 9.94bc | 127.45 ± 0.99bc | 115.63 ± 6.83bc | 154.54 ± 7.82a | 140.38 ± 11.28ab |
| *manB* | 207.52 ± 8.27b | 253.34 ± 2.37a | 218.10 ± 8.09b | 225.56 ± 6.19b | 226.40 ± 7.55b | 230.45 ± 8.81ab |
| *xylA* | 122.52 ± 4.00a | 133.90 ± 11.89a | 119.48 ± 4.05a | 130.15 ± 4.06a | 129.47 ± 3.11a | 128.57 ± 7.13a |
| *lig* | 33.20 ± 6.32a | 34.00 ± 2.13a | 34.46 ± 2.99a | 30.69 ± 0.22a | 44.00 ± 8.58a | 36.77 ± 2.44a |

Values are means of 3 replicates ± SD followed by different letters differ significantly (Turkey's honestly significant difference test, *P* < 0.05). MS, *M. sieversii* grafted onto *M. sieversii*; HF, Hanfu grafted onto *M. sieversii*; FJ, Red Fuji grafted onto *M. sieversii.* N represents nitrogen application treatment.**Table S12.** Abundance of the nitrogen cycle functional genes in rhizosphere of apple plants under different scion varieties and nitrogen levels.

| Nitrogen cycle genes | MS | HF | FJ | MSN | HFN | FJN |
| --- | --- | --- | --- | --- | --- | --- |
| *nifD* | 0.66 ± 0.14bc | 0.23 ± 0.33c | 0.94 ± 0.68bc | 0.47 ± 0.10c | 3.42 ± 0.65a | 2.03 ± 0.27ab |
| *nifH* | 0.87 ± 0.35a | 0.92 ± 0.15a | 0.96 ± 0.25a | 0.79 ± 0.55a | 2.79 ± 1.21a | 1.83 ± 0.63a |
| *nifK* | 1.08 ± 0.48ab | 0 ± 0b | 0.72 ± 0.39ab | 0.36 ± 0.10ab | 2.28 ± 1.31a | 1.03 ± 0.17ab |
| *pmoA-amoA* | 4.18 ± 0.58a | 4.09 ± 1.29a | 5.29 ± 2.23a | 4.94 ± 0.26a | 2.64 ± 0.51a | 3.62 ± 0.96a |
| *pmoB-amoB* | 5.68 ± 0.54a | 4.76 ± 0.75a | 5.45 ± 2.25a | 5.19 ± 1.32a | 3.39 ± 0.49a | 3.42 ± 0.31a |
| *pmoC-amoC* | 9.97 ± 0.56a | 9.29 ± 0.57a | 9.89 ± 2.97a | 9.06 ± 1.37a | 7.92 ± 0.63a | 7.97 ± 1.04a |
| *hao* | 5.89 ± 0.71a | 4.97 ± 1.45a | 6.17 ± 2.81a | 4.59 ± 0.80a | 5.72 ± 0.66a | 4.94 ± 0.45a |
| *narG/nxrA* | 163.21 ± 6.94a | 151.08 ± 7.68a | 157.34 ± 13.25a | 155.76 ± 9.35a | 169.94 ± 13.34a | 167.73 ± 9.11a |
| *narH/nxrB* | 77.49 ± 0.84a | 63.95 ± 4.62a | 70.71 ± 6.40a | 73.55 ± 5.56a | 77.71 ± 5.21a | 75.16 ± 2.74a |
| *narl* | 17.81 ± 2.35a | 17.98 ± 1.18a | 17.42 ± 3.04a | 17.26 ± 1.44a | 19.58 ± 1.77a | 19.88 ± 1.84a |
| *narJ* | 22.71 ± 0.70a | 18.27 ± 1.54a | 20.76 ± 3.07a | 17.51 ± 1.91a | 22.46 ± 1.76a | 21.51 ± 0.71a |
| *napA* | 79.57 ± 4.61ab | 75.42 ± 1.83b | 88.43 ± 3.67a | 71.42 ± 3.34b | 82.03 ± 3.00ab | 80.72 ± 5.33ab |
| *napB* | 19.40 ± 0.26a | 17.59 ± 3.34a | 20.16 ± 2.19a | 16.22 ± 1.04a | 18.07 ± 1.07a | 18.08 ± 2.25a |
| *napC* | 31.06 ± 3.65ab | 23.65 ± 2.72b | 33.89 ± 3.21a | 25.78 ± 2.87ab | 24.85 ± 1.48ab | 31.92 ± 1.52ab |
| *nirK* | 150.59 ± 2.60bc | 178.64 ± 6.14a | 137.26 ± 5.13c | 149.97 ± 3.39bc | 164.37 ± 10.29ab | 160.96 ± 9.37ab |
| *nirS* | 18.73 ± 3.63a | 13.19 ± 1.90a | 17.78 ± 1.50a | 14.46 ± 0.62a | 19.94 ± 2.66a | 20.48 ± 4.96a |
| *norB* | 156.67 ± 2.16c | 169.37 ± 3.14bc | 167.20 ± 12.20bc | 148.44 ± 2.17c | 205.47 ± 8.93a | 181.36 ± 3.90b |
| *norC* | 18.55 ± 1.01a | 15.44 ± 2.45a | 15.72 ± 0.96a | 16.61 ± 1.73a | 20.01 ± 2.04a | 16.30 ± 2.17a |
| *nosZ* | 99.58 ± 2.27a | 97.11 ± 3.56a | 91.11 ± 5.28a | 103.04 ± 8.63a | 100.17 ± 3.91a | 95.50 ± 7.57a |

Values are means of 3 replicates ± SD followed by different letters differ significantly (Turkey's honestly significant difference test, *P* < 0.05). MS, *M. sieversii* grafted onto *M. sieversii*; HF, Hanfu grafted onto *M. sieversii*; FJ, Red Fuji grafted onto *M. sieversii.* N represents nitrogen application treatment.

**Table S13.** The variance is explained by different environmental factors based on redundancy analysis.

| **Soil Microbes** | **Factors** | **Variance**  **explained (%)** | **ANOVA** | |
| --- | --- | --- | --- | --- |
|  |  |  | F | P |
| **Bacterial genera** | SOC | 6.5 | 2.5 | 0.082 |
|  | TN | 8.5 | 1.6 | 0.192 |
|  | SOC/TN | 3.4 | 1.5 | 0.216 |
|  | DOC | 9.6 | 2.2 | 0.100 |
|  | TDN | 15.1 | 3.2 | 0.024 |
|  | DOC/TDN | 10.8 | 1.9 | 0.110 |
|  | MBC | 9.9 | 2.8 | 0.068 |
|  | MBN | 1.3 | 0.6 | 0.672 |
|  | MBC/MBN | 6.7 | 1.6 | 0.148 |
|  | S_SC | 9.1 | 3.0 | 0.042 |
|  | S_UE | 4.4 | 1.8 | 0.098 |
| **Fungal genera** | SOC | 4.2 | 1.4 | 0.220 |
|  | TN | 8.2 | 1.7 | 0.190 |
|  | SOC/TN | 1.3 | 0.4 | 0.784 |
|  | DOC | 6.6 | 1.4 | 0.214 |
|  | TDN | 15.3 | 2.9 | 0.060 |
|  | DOC/TDN | 5.9 | 1.3 | 0.250 |
|  | MBC | 15.9 | 4.9 | 0.022 |
|  | MBN | 1.6 | 0.5 | 0.724 |
|  | MBC/MBN | 6.7 | 1.5 | 0.196 |
|  | S_SC | 8.9 | 1.8 | 0.140 |
|  | S_UE | 4.8 | 1.6 | 0.190 |
| **Carbon cycle genes** | SOC | 3.9 | 0.8 | 0.648 |
|  | TN | 7.8 | 1.6 | 0.138 |
|  | SOC/TN | 7.0 | 1.4 | 0.222 |
|  | DOC | 3.9 | 0.8 | 0.602 |
|  | TDN | 2.7 | 0.5 | 0.802 |
|  | DOC/TDN | 2.8 | 0.5 | 0.798 |
|  | MBC | 6.0 | 1.2 | 0.288 |
|  | MBN | 15.0 | 2.8 | 0.014 |
|  | MBC/MBN | 2.8 | 0.5 | 0.846 |
|  | S_SC | 7.7 | 1.5 | 0.160 |
|  | S_UE | 4.6 | 0.9 | 0.480 |
| **Nitrogen cycle genes** | SOC | 3.7 | 0.9 | 0.530 |
|  | TN | 4.2 | 1.1 | 0.376 |
|  | SOC/TN | 3.5 | 0.8 | 0.558 |
|  | DOC | 3.1 | 0.8 | 0.554 |
|  | TDN | 6.4 | 1.6 | 0.130 |
|  | DOC/TDN | 3.1 | 0.7 | 0.594 |
|  | MBC | 1.7 | 0.4 | 0.858 |
|  | MBN | 8.2 | 2.0 | 0.042 |
|  | MBC/MBN | 3.5 | 0.9 | 0.526 |
|  | S_SC | 31.0 | 7.2 | 0.002 |
|  | S_UE | 3.7 | 0.9 | 0.472 |

Abbreviations: SOM, soil organic matter; TN, total nitrogen; DOC, dissolved organic carbon; TDN, total dissolved nitrogen; MBC, microbial biomass carbon; MBN, microbial biomass nitrogen; S_SC, soil sucrase; S_UE, soil urease.
